# Neurodegenerative diseases and immune system: From pathogenic mechanism to therapy

**DOI:** 10.4103/NRR.NRR-D-25-00274

**Published:** 2025-09-03

**Authors:** Yun Chen, Ping Yin, Qianqian Chen, Yangyang Zhang, Yangyi Tang, Weifeng Jin, Li Yu

**Affiliations:** 1 The Fourth School of Clinical Medicine, Zhejiang Chinese Medical University, Hangzhou First People’s Hospital, Hangzhou, Zhejiang Province, China;; 2 School of Basic Medical Sciences, Zhejiang Chinese Medical University, Hangzhou, Zhejiang Province, China; 3 School of Pharmaceutical Sciences, Zhejiang Chinese Medical University, Hangzhou, Zhejiang Province, China; 4 The First School of Clinical Medicine, Zhejiang Chinese Medical University, Hangzhou, Zhejiang Province, China; 5 Key Laboratory of Drug Safety Evaluation and Research of Zhejiang Province, Center of Safety Evaluation and Research, Hangzhou Medical College, Hangzhou, Zhejiang Province, China

**Keywords:** Alzheimer’s disease, amyotrophic lateral sclerosis, immune system, multiple sclerosis, neurodegenerative diseases, Parkinson’s disease, pathogenic mechanism

## Abstract

Neurodegenerative diseases are a class of disorders with the gradual loss of the central nervous system and peripheral nervous system. Neurodegenerative diseases manifest primarily as cognitive and behavioral disorders that adversely affect the lives of millions of people worldwide. Therefore, it is necessary to elucidate the mechanism of neurodegenerative diseases further and find effective new therapies. In recent years, increasing evidence has shown that the immune system plays a significant role in the pathophysiology of neurodegenerative diseases and regulates this process. The central and peripheral immune systems exert different roles in the disease progression. The development of neurodegenerative diseases is influenced by interactions between them. This review focuses on how the immune system, including microglia mediated nucleotide-binding oligomerization domain-like receptor protein 3 inflammation activation and T cell-mediated neuroinflammation, interactions with neurodegenerative diseases by modulating protein aggregation and blood–brain barrier permeability. Besides, we gave particular attention to glial cell-centered multicellular interactions and the inflammatory signaling pathway. Insight into the immune system’s functions and cellular interactions is essential for progressing disease research. In addition, the functions and mechanisms of these immune cells also suggest new ideas and targets for treatment. Therefore, this review summarizes some of the existing treatment strategies for amyloid-beta, tau, neuroinflammation, α-synuclein, associated microbiota, immune modulation, and neural injury repair. In addition, this review summarizes and compares animal models of different common neurodegenerative diseases and clinical research progress. In view of the current research status, new research directions and suggestions are proposed.

## Introduction

**Figure 1:**
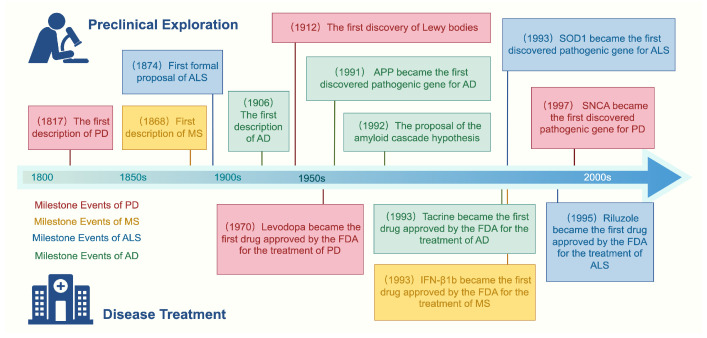
Milestone events of neurodegenerative diseases. Created with Figdraw (www.figdraw.com, ID: SYITP0e1f0). AD: Alzheimer’s disease; ALS: amyotrophic lateral sclerosis; APP: amyloid beta precursor protein; FDA: U.S. Food and Drug Administration; IFN-β: interferon-beta; MS: multiple sclerosis; PD: Parkinson’s disease; SNCA: synuclein alpha; SOD1: superoxide dismutase 1.

Since James Parkinson first detailed Parkinson’s disease (PD) in his book *An Essay on the Shaking Palsy* in 1817, neurodegenerative diseases (NDDs) have gradually become known by public (Kulcsarova et al., 2024; **Figure 1**). NDDs are a type of neurological disease with progressive loss of neurons in the central nervous system (CNS) or peripheral nervous system (PNS), resulting in cognitive and behavioral impairment in patients (Sharma et al., 2022). NDDs cover a wide range of disease types, including Alzheimer’s disease (AD), PD, multiple sclerosis (MS), and amyotrophic lateral sclerosis (ALS). With the increasing global elderly population, the number of individuals affected by NDDs has increased significantly (Üremiş and Üremiş, 2025), placing an economic and medical burden on patients, families, and society.

There are various ways to classify NDDs, which can be categorized based on predominant clinical features, anatomical distribution of neurodegeneration, or major molecular abnormalities (Dugger and Dickson, 2017). The main pathological hallmarks of NDDs include the following eight points: accumulation of pathological protein, dysfunction of synaptic and neuronal network, abnormal proteostasis, cytoskeletal abnormalities, abnormal energy metabolism, DNA/RNA abnormalities, inflammation, and neuronal cell death (Wilson et al., 2023). Recently, some experts and scholars have proposed to defining NDDs using this set of characteristic hallmarks (Wilson et al., 2023).

NDDs are driven by complex pathological mechanisms, and the pathogenesis and etiology of different diseases are different, such as protein aggregation, demyelination, ischemia (Stephenson et al., 2018). However, immune system activation and neuroinflammation involvement are common characteristics of NDDs (Stephenson et al., 2018). Neuroinflammation, which is the inflammation happening in the nervous system, is triggered by neurons, central immune cells, and peripheral immune cells in response to various inflammatory signals (Isik et al., 2023). Although some of the NDDs may have initially been considered purely neurodegenerative, clear evidence of immunity and neuroinflammation has been observed in subsequent studies and experiments in NDDs, including AD (Trojanowski, 2002; Makin, 2018), PD (McGeer et al., 1988; Yacoubian et al., 2023), MS (Brint et al., 2024; Theophanous et al., 2024), ALS (Zhang et al., 2023c). Moreover, many NDDs are associated with autoimmune system disorders, and there is even discussion about whether they should be considered as autoimmune diseases (Bandres-Ciga and Cookson, 2017; Li et al., 2023; Weaver, 2023). Microglia and astrocytes have also attracted attention in recent years and are now considered to be an important and relevant part of NDDs (Chowen and Garcia-Segura, 2021; Afridi et al., 2022; Theophanous et al., 2024). Compared to the central immune system, research on the peripheral immune system is limited, but experimental evidence found in studies reveals the involvement of the peripheral immune system (Sulzer et al., 2017; Liu et al., 2022a; Shi et al., 2022a; Theophanous et al., 2024), particularly T cells.

Thus, the immune system is essential to the progression of the NDDs and the interaction between them deserves further study. This review focuses on AD, PD, MS, ALS, summarizes and discusses the pathogenic mechanism, treatment strategy, clinical research progress, research methods and technical progress of the immune system in NDDs.

## Search Strategy

This study searched PubMed for this review by mainly using keywords such as “neurodegenerative diseases,” “Alzheimer’s disease,” “Parkinson’s disease,” “multiple sclerosis,” “amyotrophic lateral sclerosis,” “genetics,” “neuroinflammation,” “signaling pathways,” “immune system,” “microglia,” “T cells,” “B cells,” “treatment,” “microbiota,” “immunomodulation,” “neural repair,” “animal model,”“multi-omics,” “genomics,” “transcriptomics,” “proteomics,” “metabolomics,” “genome-wide association studies.” These keywords were combined in various ways to search the literature without any date restrictions. For the part of clinical trails, ClinicalTrials.gov was searched with the following keywords: “Condition/Disease” column: “Alzheimer’s disease” OR “Parkinson’s disease” OR “multiple sclerosis” OR “amyotrophic lateral sclerosis;” “Intervention/treatment” column: “antibody” OR “vaccine” OR “inflammation”; “Study Phase”column: “Phase 1” OR “Phase 2” OR “Phase 3;” “Study Type” column: “Interventional.” Only those studies that were considered most relevant to the core topic of this review were selected for inclusion.

## Pathogenic Mechanism of Neurodegenerative Diseases

### Genetic evidence

Previous evidence has demonstrated that genetic factors are central to the etiology of NDDs, providing new insights into NDD development (Pihlstrøm et al., 2017).

#### Genetic evidence of Alzheimer’s disease

One common type of NDD that primarily impacts the elderly is AD. Aggregated amyloid-beta (Aβ), intracellular neurofibrillary tangles, and misfolded microtubule-associated protein tau (tau) are the classic indicators of AD.

The amyloid hypothesis believes that the protein accumulation of Aβ has a number of downstream effects and is the key cellular driver influencing the course of AD, ultimately resulting in dementia and cognitive dysfunction (Hardy and Higgins, 1992; Selkoe and Hardy, 2016). Data suggests that AD appears to be one of the most genetically influenced human multifactorial diseases, with heritability estimated ranging from 58%–79% (Gatz et al., 2006). AD is classified into two categories: late-onset AD and early-onset AD. It can also be classified into familial AD (FAD) and sporadic AD.

Three known types of FAD recognized so far are all autosomal dominant, typically occurring in families with reports of early onset (before 65 years old). Such cases account for only 1% of all cases. Based on available literature, three gene mutations have been connected to autosomal dominant types of FAD: amyloid beta precursor protein (*APP*), presenilin 1 (*PSEN1*) (Levy-Lahad et al., 1995) and presenilin 2 (*PSEN2*) (Sherrington et al., 1995). *APP* is located on chromosome 21 and is expressed in various tissues, but primarily in neurons of the CNS (Niranjan, 2013). APP, as a transmembrane protein transported via secretory and endocytic pathways, can be cleaved by β-secretase and γ-secretase (Sehar et al., 2022). PSEN1 and PSEN2 are part of the γ-secretase complex and are involved in various physiological processes, including the Notch signaling pathway, which regulates immune cell function (Croft et al., 2022; Wolfe and Miao, 2022).

The main causes of FAD include Aβ production, the Aβ_42_/Aβ_40_ ratio, or the Aβ structure (Holtzman et al., 2011; Shi and Holtzman, 2018). Clinically, the majority of AD patients are late-onset sporadic forms; these cases typically begin in adults over 65 and do not appear to be familially clustered. Genome-wide association studies (GWAS) have gradually revealed genes or loci linked to AD. In one study, 75 risk genes for AD were identified through GWAS (Bellenguez et al., 2022). Among these risk genes, many are involved in immune cell function, particularly including microglial and the function of innate immune cell, including apolipoprotein E (*APOE*), triggering receptor expressed on myeloid cells 2 (*TREM2*), ATP binding cassette subfamily A member 7 (*ABCA7*) and complement C3b/C4b receptor 1 (*CR1*) (Bellenguez et al., 2022; Kloske et al., 2023). *APOE* is the strongest genetic risk factor for late-onset AD. It is currently believed that the *ε4* allele significantly increases the risk of disease, but the *ε2* allele appears to be protective (Raulin et al., 2022). ApoE binds to Aβ receptors in a competitive manner, including low-density lipoprotein receptor-related protein 1 (LRP1), therefore blocking the uptake of Aβ and thereby affecting Aβ clearance (Long and Holtzman, 2019). Compared with *APOE-ε3/3*-AD individuals, *APOE-ε4/4*-AD individuals have impaired inflammatory pathways and significant differences in key pathways (e.g., microglial function, epigenetic regulation, oligodendrocyte function) (Kloske et al., 2021). There are other AD-associated immune risk genes, such as *CD33*, membrane spanning 4-domains A6A (*MS4A6A*), inositol polyphosphate-5-phosphatase D (*INPP5D*), human leukocyte antigen (*HLA*), myocyte enhancer factor 2C (*MEF2C*), ephrin type-A receptor 1 (*EPHA1*) and protein tyrosine kinase 2 beta (*PTK2B*) (Eid et al., 2019). In addition, a team found that the genetic architecture of AD differs between Chinese and European populations, and found that *KIAA2013*, solute carrier family 52 member 3 (*SLC52A3*), and transcobalamin 2 (*TCN2*) were associated with AD in the Chinese population (Ge, Chen, et al., 2024). This finding is of great significance for studying the population-specific genetic mechanisms of the disease.

#### Genetic evidence of Parkinson’s disease

The main pathological basis of PD is the degeneration of dopaminergic neurons (DA neurons) in the substantia nigra (SN) compacta and is characterized by aggregates of α-synuclein (α-syn) (Torii et al., 2023). The main symptoms of PD are motor dysfunction, such as rigidity, tremor, bradykinesia, and postural instability. The heritability of PD is about 27% overall, suggesting that behavioral and environmental factors have a greater influence, especially late-onset PD (Warner and Schapira, 2003; Goldman et al., 2019). To date, 90 independent variants from 74 genomic loci have been found to be linked to PD risk (Morris et al., 2024). Each variant increases the risk of disease slightly, with odds ratios of less than 2 (Morris et al., 2024). Thus, although a genetic form of PD has been identified, most cases are sporadic and there is no known exact cause (Costa et al., 2023). GWAS-identified risk variants may be related to gene expression or alternative splicing. Gene-level analysis of expression revealed that the regulation of WD repeat domain 6 (*WDR6*), *CD38*, glycoprotein nonmetastatic melanoma protein B (*GPNMB*), Ras-related protein Rab-29 (*RAB29*), and transmembrane protein 163 (*TMEM163*) expression is associated with PD (Kia et al., 2021). Moreover, compared to neurons, the expression of colocalization analysis prioritized genes is more prevalent in glial cells (Kia et al., 2021). Zinc finger RANBP2-type containing 3 (*ZRANB3*), polycomb group ring finger 3 (*PCGF3*), never in mitosis gene A related kinase 1 (*NEK1*), nucleoporin like 2 (*NUPL2*), galactosylceramidase (*GALC*), and cathepsin B (*CTSB*) have been shown to be associated with splicing variation (Kia et al., 2021). Of all cases, only 5%–10% are familial PD (Funayama et al., 2023). Seven genes have currently been found to be monogenic causes of familial PD (Lange et al., 2022): four genes, including leucine rich repeat kinase 2 (*LRRK2*), coiled-coil-helix-coiled-coil-helix domain containing 2 (*CHCHD2*), vacuolar protein sorting-associated protein 35 (*VPS35*), and synuclein alpha (*SNCA*), cause late-onset, autosomal dominant inheritance; while three genes, including Parkin RBR E3 ubiquitin-protein ligase (*PARKIN*), parkinsonism associated deglycase (*DJ-1*), and PTEN-induced kinase 1 (*PINK1*), cause early-onset, autosomal recessive inheritance (Ben-Shlomo et al., 2024). Besides, variation in glucocerebrosidase (*GBA*) is the most prevalent genetic factor (Ben-Shlomo et al., 2024). The genes associated with monogenic PD have potential roles in regulating immune responses: with the exception of *CHCHD2*, all of these genes have been reported in the literature (**Table 1**) to be involved in immune regulation or associated with the immune response; *CHCHD2* has been found to be potentially associated with inflammatory responses under specific conditions (Cheng et al., 2019; Bosco et al., 2023).

**Table 1:** The association between single gene Parkinson’s disease-related genes and the immune system

Gene	Relationship with the immune system	Reference
*LRRK2*	*LRRK2* expression influences or regulates inflammatory response, autophagy, cytokine release, and phagocytosis.	Russo et al., 2022; Yan et al., 2022; Wallings et al., 2023
*CHCHD2*	The systemic inflammatory state of full-term clinical chorioamnionitis promotes elevated *CHCHD2/MNRR1* in maternal blood.	Cheng et al., 2019; Bosco et al., 2023
*VPS35*	*VPS35* supports TREM2 recycling and stability, thereby inhibiting microglial overactivation and limiting the overproduction of inducible nitric oxide synthase and interleukin-6. Loss of *VPS35* or R47H mutation leads to TREM2 degradation and exacerbates inflammation.	Yin et al., 2016; Appel et al., 2018
*SNCA*	It is involved in regulating microglia phenotypic changes, phagocytosis, cytokine secretion, and is involved in immune cell infiltration in cancer.	Austin et al., 2011; Aloy et al., 2024; Rajasekaran et al., 2024
*PARKIN*	*PARKIN* has an inhibitory effect on innate immunity. In cancer, it activates innate immunity and promotes anti-tumor immune responses. The absence of PARKIN promotes neuroinflammation.	Matheoud et al., 2016; Sliter et al., 2018; Perego et al., 2024
*DJ-1*	It is involved in immune regulation, regulates the activation of a variety of immune cells (including microglia, macrophages, mast cells, and T cells), triggers inflammatory responses, and regulates the activation of NLRP3 inflammasomes.	Zhang et al., 2020a; Nakamura et al., 2021; Cai et al., 2022; Zeng et al., 2022b
*PINK1*	Compared with the control group, more macrophage infiltration and glial cell overactivation were observed in the spinal cord of *PINK1* gene knockout mice. In vitro experiments, lack of *PINK1* causes abnormal inflammatory responses in microglia and astrocytes.	Sun et al., 2018; Cossu et al., 2022
*GBA*	*GBA* regulates immune cell activation, development, recruitment, and antigen presentation. Mutations cause elevated levels of pro-inflammatory cytokines, microglia activation, and astrocyte proliferation.	Liu et al., 2012; Usenko et al., 2021; Cossu et al., 2023

CHCHD2: Coiled-coil-helix-coiled-coil-helix domain containing 2; DJ-1: parkinsonism associated deglycase; GBA: glucocerebrosidase; LRRK2: leucine rich repeat kinase 2; PARKIN: Parkin RBR E3 ubiquitin-protein ligase; PINK1: PTEN-induced kinase 1; SNCA: synuclein alpha; VPS35: vacuolar protein sorting-associated protein 35.

#### Genetic evidence of multiple sclerosis

MS is considered an autoimmune disease of the CNS that can cause both acute inflammatory lesions and chronic inflammatory responses. Although the precise trigger of MS is unknown, it is thought to be caused by the interplay of genetic predisposition and environmental influences. Inflammatory states and nerve damage may underlie the pathology (Cocchi et al., 2022). Its hereditary nature is now mostly explained and is almost entirely due to genes that influence the immune response (Parnell and Booth, 2017). Studies in Sweden (Westerlind et al., 2014) and Italy (Boles et al., 2023) suggested that the heritability of MS is in the range of 48%–64% (Kim and Patsopoulos, 2022).

The first genetic associations in MS were within the HLA gene cluster (Bertrams et al., 1972). The strongest known genetic risk factor to date is the HLA-DRB1*15:01 allele, located in the major histocompatibility complex (MHC) class II region (Hollenbach and Oksenberg, 2015). Located on the short arm of chromosome 6, the gene’s main function is to present antigens to CD4^+^ T cells, contributing to the regulation of adaptive immune responses (Montgomery et al., 2024). This allele is detected in approximately 60% of Caucasian MS patients and leads to an approximately threefold elevated risk of developing MS in carriers (Greer, 2015; Barrie et al., 2024). However, HLA variants account for only a part of the genetic risk. Based on GWAS research, more than 200 non-HLA susceptibility loci have been further identified, most of which are related to immune cells and enriched in CD4^+^/CD8^+^ T cells, B cells, natural killer (NK) cells, microglia, and dendritic cells, but not in astrocytes or neurons (International Multiple Sclerosis Genetics Consortium, 2019). These susceptibility genes are involved in multiple immune regulatory pathways, such as T cell co-stimulation, including *CD40* and *CD28*; interleukin signaling, including interleukin 2 receptor subunit alpha (*IL2RA*) and interleukin 7 receptor (*IL7R*); type I interferon response, including tyrosine kinase 2 (*TYK2*) and signal transducer and activator of transcription 3 (*STAT3*); and the nuclear factor kappa-B (*NF-κB*) pathway, such as tumor necrosis factor receptor superfamily member 1A (*TNFRSF1A*) (De Jager et al., 2009; Sawcer et al., 2011; Mahurkar et al., 2013).

In addition, a previous study has found that family members of MS patients have an increased risk of developing other autoimmune diseases (Kim and Patsopoulos, 2022). There is genetic overlap between MS and other autoimmune diseases such as type 1 diabetes, rheumatoid arthritis, and systemic lupus erythematosus (Cotsapas et al., 2011). Some genetic variants associated with MS show resistance to various pathogens (Barrie et al., 2024). For example, HLA-DRB1*15:01 shows a protection against tuberculosis but increases the risk of leprosy (Barrie et al., 2024).

#### Genetic evidence of amyotrophic lateral sclerosis

As a motor neuron (MN) disease, ALS involves the progressive loss of both upper and lower MNs, which can result in cognitive and behavioral dysfunction. Clinically, it is also classified as familial ALS (fALS) and sporadic ALS (sALS). The family history of ALS or frontotemporal dementia (FTD) is present in 10%–15% of patients, and autosomal dominance is typically the mode of inheritance for fALS (Alsultan et al., 2016). 85%–90% of patients have no known family history and usually do not carry genes with known rare and highly penetrant mutation (Gregory et al., 2020; Goutman et al., 2022).

The genetic structure of ALS is very complex and is mainly based on single-gene inheritance of rare variants (Feldman et al., 2022). To date, more than 40 related genes have been identified, and the most prevalent and penetrant mutations are found in chromosome 9 open reading frame 72 (*C9ORF72*), TAR DNA-binding protein (*TARDBP*), superoxide dismutase 1 (*SOD1*), and fused in sarcoma (*FUS*) (Feldman et al., 2022). About 5% of all ALS cases are caused by abnormalities in the *TARDBP* and fused in sarcoma/translocated in liposarcoma (*FUS/TLS*) genes; 15%–20% of fALS cases are caused by *SOD1* mutations; and 30%–40% of fALS cases are caused by *C9ORF72* gene mutations (Volonté et al., 2019). Other Mendelian genes associated with ALS include neurofilament heavy chain (*NEFH*), Alsin Rho guanine nucleotide exchange factor (*ALS2*), dynactin subunit 1 (*DCTN1*), vesicle associated membrane protein associated protein B (*VAPB*), senataxin (*SETX*), charged multivesicular body protein 2B (*CHMP2B*), valosin containing protein (*VCP*), spastic paraplegia 11 (*SPG11*), optineurin (*OPTN*), ubiquilin 2 (*UBQLN2*), sequestosome 1 (*SQSTM1*), profilin 1 (*PFN1*), heterogeneous nuclear ribonucleoprotein A2/B1 (*HNRNPA2B1*), heterogeneous nuclear ribonucleoprotein A1 (*HNRNPA1*), tubulin alpha 4a (*TUBA4A*), matrin 3 (*MATR3*) (Akçimen et al., 2023). It is worth noting that some of these ALS-related genes are functionally involved in the regulation of the immune system, indicating an immunogenetic background. For instance, it was discovered in animal experiments that the mutation of *C9ORF72* induces excessive activation of T cells by influencing autophagy and inflammatory regulatory pathways in macrophages and microglia, including upregulating the expression of the co-stimulatory molecule CD80 (Cicardi and Trotti, 2024). *OPTN*, *SQSTM1*, and *TBK1* play a role in the NF-κB signaling pathway (Cirulli et al., 2015). However, γ-aminobutyric acid neurons and oligodendrocytes are found significantly enriched in ALS risk, rather than microglia or other non-neuronal cell types (Saez-Atienzar et al., 2021; van Rheenen et al., 2021). This suggests that the immune system plays a role in ALS, but that role is limited. According to twin studies, the heritability of sALS is approximately 60%, indicating the importance of genetic factors (Al-Chalabi et al., 2010; Rizea et al., 2024). About 8% of sALS cases and 40% of fALS cases are caused by *C9ORF72* repeat expansion, which is the primary genetic factor for ALS and FTD (Rizea et al., 2024).

In addition, there is a genetic correlation between ALS and autoimmune diseases. There is a significant positive genetic correlation between celiac disease, rheumatoid arthritis, systemic lupus erythematosus, and ALS (Li et al., 2021a). Mendelian randomization studies have identified potential causal relationships between ALS and six different types of immune cells (Tang et al., 2024). Besides, an epigenome-wide association study found that ALS samples were enriched in multiple immune-related traits, including IgE levels and allergy sensitivity.

### Immune cells and inflammation in neurodegenerative diseases

#### Interplay between the immunity cells, inflammation, and Alzheimer’s disease

*The central role of microglia*

Human genetic data indicate that microglia play a central role in the pathogenesis of AD (Hansen et al., 2018). Microglia are macrophages in the human brain that serve a variety of functions, including the innate immune response. To keep the body’s metabolic homeostasis intact, microglia have the ability to phagocytose errant proteins such as Aβ and cellular debris (Sarlus and Heneka, 2017). In normal brain, microglia are predominantly in a resting state (M0). Numerous stimuli can activate microglia, such as invading harmful substances (e.g., lipopolysaccharides), misfolded proteins (e.g., Aβ), and brain damage (Willis et al., 2020; Merighi et al., 2022). Activated microglia generally fall into two categories: M1 (pro-inflammatory) or M2 (anti-inflammatory). Switching between these two states is associated with remyelination (Kwon and Koh, 2020), and an imbalance between the two states exacerbates the CNS disease (Guo et al., 2022). Additionally, the percentage of each phenotype may vary based on the NDD stage (Tang and Le, 2016). Research has indicated that the recruited microglia at the site of injury initially exhibit the M2 phenotype before progressively switching to the M1 phenotype approximately a week later, leading to an exacerbation of the inflammatory response (Wu and Eisel, 2023). M1 activation, also referred to as classical activation, is typically induced by lipopolysaccharide and interferon-γ (IFN-γ) (Guo et al., 2022). Peripheral neurons may be damaged by inducible nitric oxide synthase’s ability to upregulate inflammatory pathways and encourage the release of numerous inflammatory factors, including tumor necrosis factor-α (TNF-α), interleukin-6 (IL-6), IL-1β, and nitric oxide. Anti-inflammatory cytokines such as IL-4 and IL-13 may result in M2 activation, also referred to as alternative activation, which exhibits anti-inflammatory and protective properties. It is characterized by the release of anti-inflammatory cytokines including transforming growth factor-β (TGF-β), IL-4, IL-10, and IL-13 (Colonna and Butovsky, 2017).

**Figure 2:**
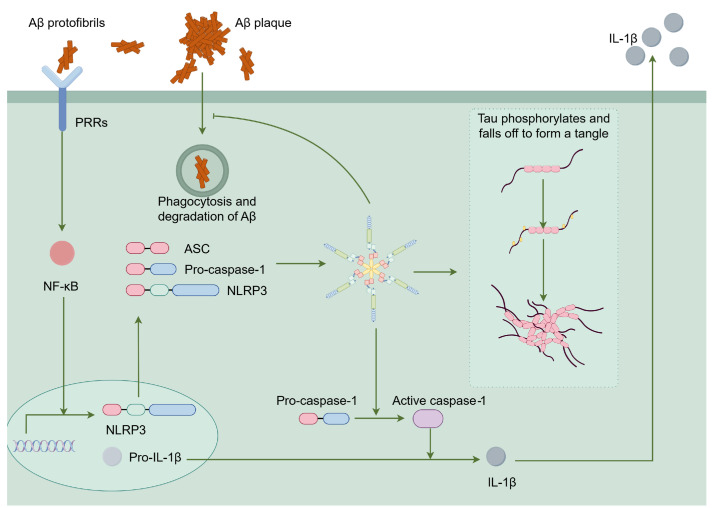
Schematic diagram of the NLRP3 pathway within microglia. Microglia induce inflammatory response signaling pathways through pattern recognition receptors. At the same time, it activates NLRP3 inflammasomes, increases pro-inflammatory factors, inhibits the phagocytosis of microglia, and promotes AD pathology. Created with Figdraw (www.figdraw.com, ID: IOTWO2333a). AD: Alzheimer’s disease; ASC: adaptor apoptosis-associated speck-like protein containing a CARD; Aβ: amyloid-beta; IL-1β: interleukin-1 beta; NF-κB: nuclear factor kappa-B; NLRP3: nucleotide-binding oligomerization domain-like receptor protein 3; PRRs: pattern recognition receptors.

In early AD, microglia normally gather close to Aβ (Stalder et al., 1999) and uptake amyloid through phagocytosis, pinocytosis, and receptor-dependent endocytosis (Nakaso, 2024). However, the balance between Aβ accumulation and clearance is disrupted and chronic inflammation develops once the concentration of Aβ rises to a certain point. Unlike the acute response phase, chronic inflammation occurs with the release of large amounts of cytokines, a process that is often detrimental to the brain and leads to Aβ aggregation and the development of AD pathology (Kim and Joh, 2006; Kinney et al., 2018). When the soluble Aβ oligomers and fibrils bind to receptors on microglia, inflammatory cytokines and chemokines will be released, which will worsen neuroinflammation (Ma et al., 2017). Furthermore, genes involved in Aβ clearance are downregulated by pro-inflammatory cytokines generated in response to Aβ deposition, which encourages Aβ buildup and neurodegeneration (Hickman et al., 2008). As the disease progresses, microglia will be difficult or unable to clear Aβ (Kinney et al., 2018), so peripheral immune cells may be recruited into Aβ plaque deposition to clear Aβ, leading to further inflammation (Jay et al., 2015). Exacerbated inflammation further reduces the ability of microglia to clear, thus causing a vicious circle. The intrinsic mechanism of microglia involvement in AD is related to the inflammasome: microglia sense various pathogen-associated molecular patterns and damage-associated molecular patterns through pattern recognition receptors, promote signaling pathways to induce inflammatory responses, and exert phagocytosis to clear Aβ (Friker et al., 2020). At the same time, it activates nucleotide-binding oligomerization domain-like receptor protein 3 (NLRP3) inflammasomes. On one hand, NLRP3 activation promotes the conversion of pro-caspase-1 to active caspase-1, resulting in an increase in pro-inflammatory factors (Voet et al., 2019). On the other hand, it reduces the ability of microglia to phagocytose Aβ, thus increasing Aβ deposition and worsening the condition (Heneka et al., 2013). NLRP3 is critical in the pathogenesis of AD. According to an experimental study, in the APP/PS1 model of AD, NLRP3 inflammasome defects bias microglia towards the M2 phenotype and results in reduced Aβ deposition (Heneka et al., 2013). Furthermore, activation of the NLRP3 inflammasome promotes aberrant tau synthesis, leading to disease progression (Ising et al., 2019; **Figure 2**).

Immune infiltration is an important part of AD pathology, and microglia play a central role, as evidenced by their interactions and recruitment with other immune cells. The mechanism of its recruitment is complex, which may be caused by multiple reasons. As mentioned above, microglia are activated by Aβ and tau aggregates and secrete pro-inflammatory molecules. Activated microglia interact with astrocytes (Jha et al., 2019a; Chen et al., 2023), inducing astrocytes to form reactive astrocytes, causing astrocytes to secrete cytokines, chemokines, and neurotrophic factors (e.g., TGF-β) (Netzahualcoyotzi et al., 2024). It has been demonstrated that these factors directly impact the integrity of blood–brain barrier (BBB) (Netzahualcoyotzi et al., 2024). Additionally, microglia regulate endothelial cell responses to inflammatory damage, altering BBB properties. The increased cyclooxygenase-2 (COX-2) activity in activated microglia can promote the release of inflammatory cytokines (TNF-α, IL-1β, and IL-6) and lead to the destabilization of BBB (Akundi et al., 2005; Spampinato et al., 2022). Although in the short term, microglia provide some protection for the BBB, in the long term, chronic inflammation causes an increase in BBB permeability (Haruwaka et al., 2019). The pro-inflammatory cytokines produced by microglia can also promote the expression of cell adhesion molecules on pericytes. In the presence of chemokines secreted by microglia, this can promote the transport of immune cells across BBB (Persidsky et al., 2016). In addition, microglia collaborate with T and B cells to inhibit disease progression via cytokine and antibody-mediated mechanisms (Bergen et al., 2015; Su et al., 2023). An experiment that depleted microglia in APPPS1/TK mice found no significant change in the amount of Aβ deposits, even when peripheral myeloid cells were recruited to the CNS (Prokop et al., 2015), indicating that microglia play a central role in AD.

*Relationship between astrocytes and Alzheimer’s disease*

Astrocytes are another type of glial cells that play a major function in neuromodulation, and they have a star-shaped morphology. Astrocytes have been observed many physiological functions, such as release of growth factors (McAlpine et al., 2021), regulation of synapses (Brandebura et al., 2023), neuroprotection (Bélanger and Magistretti, 2009), and involvement in the formation of the BBB (Heymans et al., 2020). Astrocytes also have pro-inflammatory and neuroprotective subsets. Pro-inflammatory reactive astrocytes induce harmful cytokine release (e.g., IL-1β and TNF-α) (Kwon and Koh, 2020). Neuroprotective subsets of astrocytes are activated by anti-inflammatory cytokines (e.g., IL-4, TGF-β, and IL-10), which also encourage the release of anti-inflammatory cytokines (Kwon and Koh, 2020).

Aβ can also be cleared by astrocytes. Chemokines surrounding Aβ attract and recruit astrocytes, which subsequently decrease Aβ deposition through the receptor for advanced glycation end products (RAGE), scavenger receptor class B member 1 (SCARB1), LRP1, and lysosomes (Ling et al., 2017). Astrocytes not only remove Aβ deposits by phagocytosis (Xiao et al., 2014), but also participate in the lymphoid system-like fluid clearance pathway. In patients with AD, approximately 65% of Aβ peptide is cleared from the brain via the lymphatic system (Brandebura et al., 2023). Loss of aquaporin 4 in astrocytes results in a strongly reduced clearance via this pathway, a result suggesting that this pathway is dependent on aquaporin 4 (Xu et al., 2015). Astrocytes activated by misfolded proteins produce a variety of inflammatory cytokines. These inflammatory cytokines promote pathological changes in AD. Similar to microglia, astrocytes have an NF-κB pathway similar to microglia (Lopez-Rodriguez et al., 2021; Jung et al., 2023). Once this pathway is activated, astrocytes secrete associated inflammatory cytokines that cause neuroinflammation or lead to neural death.

Astrocytes and microglia have a certain similarity and cross-talk with each other, and the relationship between the two is very close, and their dysfunction may lead to neuroinflammation. Crosstalk between the two can be maintained by secreted mediators (e.g., cytokines, chemokines, and growth factors; Jha et al., 2019b; Chen and Yu, 2023; Wu and Eisel, 2023). In addition, communication between microglia, astrocytes, and neurons is achieved through the release and response of extracellular vesicles (e.g., exosomes and microvesicles) (Wu and Eisel, 2023). IL-1α and TNF-α secreted by microglia can induce phenotypic changes in reactive astrocytes. At the same time, IL-3 production by astrocytes can trigger changes in microglial immune response, motility, and clearance function (McAlpine et al., 2021). IL-3 has been shown to alleviate AD by modulating microglia (McAlpine et al., 2021). Astrocytes release a variety of chemokines, such as C–C motif chemokine ligand 2 (CCL2), C–X–C motif chemokine ligand 1 (CXCL1), and CXCL10, and microglia can express some corresponding chemokine receptors, including C–C chemokine receptor type 2 (CCR2) and C–X–C chemokine motif receptor 7 (Wu and Eisel, 2023).

**Figure 3:**
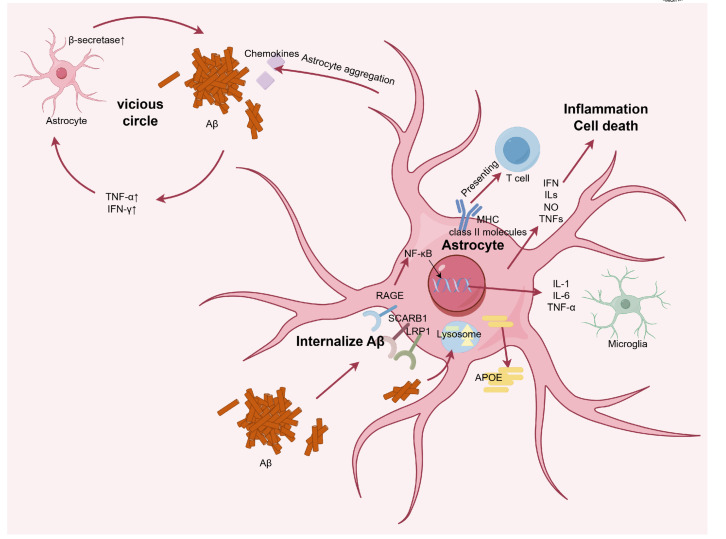
Role of astrocytes in Aβ-mediated neuroinflammation and intercellular interactions in AD. Astrocytes internalize Aβ via receptors such as RAGE, SCARB1, and LRP1, triggering NF-κB signaling and cytokine release. These responses lead to microglia and T cell activation, aggravating neuroinflammation. Besides astrocytes participate in promoting feedforward loops and accelerates Aβ pathology. Created with Figdraw (www.figdraw.com, ID: WYUSA9999b). AD: Alzheimer’s disease; APOE: apolipoprotein E; Aβ: amyloid-beta; IFN: interferon; IL: interleukin; LRP1: low-density lipoprotein receptor-related protein 1; MHC: major histocompatibility complex; NF-κB: nuclear factor kappa-B; NO: nitric oxide; RAGE: receptor for advanced glycation end products; SCARB1: scavenger receptor class B member 1; TNF: tumor necrosis factor.

The cross-talk between astrocytes and AD may be beneficial for AD patients by enhancing the degradation of Aβ aggregates (Rostami et al., 2021). However, it is also possible that crosstalk promotes disease progression (Ling et al., 2017). The study found that TNF-α and interferon-γ together stimulate the activation of astrocytes, resulting in an increase in Aβ secretion and ultimately an increase in the amount of Aβ in the brain. Astrocytes are then activated and secrete more TNF-α and IFN-γ, inducing an increase in β-secretase expression. The β-secretase in turn leads to the production of more Aβ, thus causing a vicious circle (Ling et al., 2017). Besides, in response to persistent stress, the microglia NLRP3 inflammasome can activate the neuroinflammatory caspase-1 pathway, which in turn triggers neurotoxic astrocyte activity, thereby affecting neuronal function (Li et al., 2022c; **Figure 3**).

Astrocytes can also produce complement protein C3a by Aβ-activated NF-κB signaling in microglia and aggravate Aβ pathology by promoting feedforward loops (Li et al., 2022c). Astrocytes secrete complement C3 when stimulated by inflammatory signals, which is subsequently cleaved into C3b and C3a by related enzymes. Peripheral immune cells are recruited to the brain by C3a, which further exacerbates neuroinflammation (Jevtic et al., 2017). Astrocytes also use cytokines or MHC molecules to interact with other cells, causing a range of downstream reactions (Linnerbauer et al., 2020; **Figure 3**).

*Involvement of peripheral immune cells in Alzheimer’s disease*

Despite the fact that peripheral immunity is crucial in AD (Marsh et al., 2016), it has received limited attention. Peripheral immune cells related to AD mainly include T cells, B cells, NK cells, etc (Yang et al., 2023). Immunoanalysis of AD patients revealed aberrations in the immune population that indicate heterogeneity in peripheral immune cells mentioned above (Gate et al., 2020; Brioschi et al., 2021; Lu et al., 2021). Numerous indications point to the infiltration of peripheral immune cells, such as T cells, neutrophils and NK cells, into the CNS (Baik et al., 2014; Gate et al., 2020), and the involvement of certain immune products in the pathological process of AD. Under physiological conditions, the BBB restricts lymphocyte entry into the CNS. Nevertheless, in the pathological state of AD, they can cross the BBB through multiple mechanisms and thereby trigger a series of reactions. Cerebral amyloid angiopathy, considered an early AD pathology, is a type of cerebral small vessel disease caused by Aβ deposition in the cerebral cortex and leptomeningeal small blood vessels, and is characterised by BBB leakage (Yang et al., 2023). Cytokine secretion and increased adhesion molecules induce neuroinflammation and BBB dysfunction (Persidsky et al., 2016; Yang et al., 2023). Peripheral immune cell infiltration in AD is thought to be caused directly or indirectly by BBB dysfunction (Liu et al., 2023).

• T cells

T cells, the major effector cells in cellular immunity, have three major subtypes of T cells: helper (CD4^+^ T cells), cytotoxic (CD8^+^ T cells), and regulatory (regulatory T cells, Tregs) (Schetters et al., 2018). Though low in levels, T cells play crucial roles in brain development, including maintaining brain homeostasis of development and function. The content of CD4^+^ and CD8^+^ T cells in the brain of AD patients is higher than that of healthy people (Ferretti et al., 2016; Gate et al., 2020; Jorfi et al., 2023a). The mechanism of their presence and action in AD is not yet clear, but it is currently believed to be related to the promotion or reduction of inflammation.

CD4^+^ T cells

**Figure 4:**
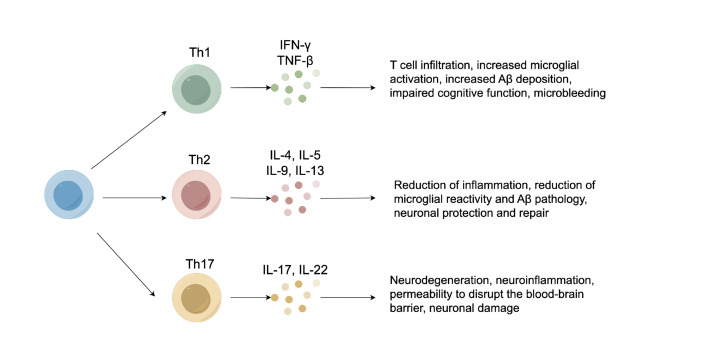
Schematic diagram of the role of CD4^+^ T cells in AD pathology. Different CD4^+^ T cells release different interleukins and perform distinct activities to help regulate the degenerative process of AD. Created with Figdraw (www.figdraw.com, ID: TWWRT61313). AD: Alzheimer’s disease; Aβ: amyloid-beta; IFN-γ: interferon-gamma; IL: interleukin; Th: T helper cells; TNF-β: tumor necrosis factor-beta.

**Figure 5:**
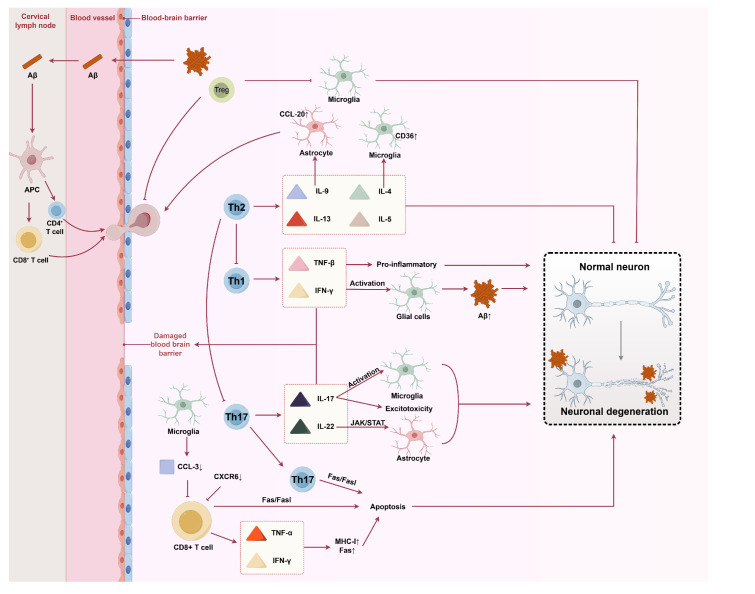
Schematic diagram of the mechanisms by which T cells are involved in AD. CD4^+^ T cells participate in the regulation process mainly by secreting cytokines. CD8^+^ T cells can induce apoptosis of target cells by secreting related substances or by FasL-Fas binding. Treg is a special type of CD4^+^ T cells, which has a certain protective effect. T cells also influence the development of neurodegeneration by interacting with microglia and astrocytes via signaling pathways (e.g., JAK/STAT). Created with Figdraw (www.figdraw.com, ID: YSOWR43536). AD: Alzheimer’s disease; APC: antigen-presenting cell; Aβ: amyloid-beta; CCL: C–C motif chemokine ligand; CXCR6: C–X–C motif chemokine receptor 6; Fas: Fas cell surface death receptor; Fasl: Fas ligand; IFN-γ: interferon-gamma; IL: interleukin; JAK/STAT: Janus kinase/signal transducer and activator of transcription; MHC: major histocompatibility complex; Th: T helper cells; TNF: tumor necrosis factor; Treg: regulatory T cell.

CD4^+^ T cells secrete a variety of cytokines, including ILs, TGF, TNF, and interferon, which regulate the Tregs and effector T cells (Teff) (Asamu et al., 2023). Activated antigen-presenting cells utilize MHC to present misfolded autoantigens to CD4^+^ T cells. Upon recognition of specific antigens, CD4^+^ T cells differentiate into various subtypes, which include Th1, Th17, and Th2 (**Figure 4**). Th1 secretes a variety of cytokines, including IFN-γ and TNF-β. The inflammatory cytokines TNF-β and IFN-γ will also cause the BBB to become more permeable and immune cell infiltration to increase. Th17 mainly secretes IL-17 and IL-22, which act synergistically on the BBB and increase its permeability, allowing peripheral lymphocytes to enter the CNS (Liu and Meng, 2023). IL-17 can promote neuroinflammation and neuronal death, as demonstrated in a human induced pluripotent stem cell-based model (Sommer et al., 2018). IL-17 triggers cognitive and synaptic defects in early AD, and when it is neutralized, symptoms in mice improve (Brigas et al., 2021; Vellecco et al., 2023). IL-22 is a potent pro-inflammatory cytokine and the regulatory mechanism involved in IL-22 may be related to the Janus kinase/signal transducer and activator of transcription (JAK/STAT) signaling pathway (Reichenbach et al., 2019; Chen et al., 2022). In addition, direct contact of Th17 with neurons leads to neuronal apoptosis. The cause of Th17-mediated neuronal death is thought to be linked to the binding of Fas ligand (FasL) on Th17 cells and Fas cell surface death receptor (Fas) on neurons (Zhang et al., 2013; **Figures 4** and **5**). Unlike Th1 and Th17, Th2 has certain protective and anti-inflammatory effects on the nervous system (Feng et al., 2022). For example, IL-4 can inhibit Aβ-induced TNF-α and IL-6 expression by astrocytes, thus alleviating neuroinflammation (Shimizu et al., 2008). In addition, IL-4 enhances microglia clearance by increasing the expression of the microglia clearance receptor CD36 (Shimizu et al., 2008). IL-13, another cytokine secreted by Th2, is also an anti-inflammatory factor that modulates inflammation.

CD8^+^ T cells

CD8^+^ T cells were found to be the predominant T cell subset infiltrating brain structures and infiltrating parenchymal brain tissue in an age- and pathology-related manner (Stojić-Vukanić et al., 2020; Unger et al., 2020). For example, there is a high concentration of effector memory CD8^+^ T cells that re-express CD45RA (TEMRA), in the brains and cerebrospinal fluid (CSF) of AD patients. TEMRA cells localize to blood vessels and accumulate adjacent to thick amyloid plaques (Gate et al., 2020). Compared to the control group, a significant increase in TEMRA cell count was found, and this increase was closely correlated with the severity of cognitive decline in patients (Gate et al., 2020). CD8^+^ T cells are implicated in both promoting and preventing AD pathology. CD8⁺ cytotoxic T cells first induce activation of the caspase cascade through FasL-Fas binding and release granzyme and perforin to promote apoptosis (Hosking et al., 2014). In addition, it can also interact with microglia, thereby alleviating or exacerbating disease progression. *In vitro* experiments, CD8^+^ T cells alone have been found to cause neuronal and/or neurite damage, and microglia working with CD8^+^ T cells to exacerbate neurodegeneration in AD cultures (Jorfi et al., 2023b). However, in another experiment, it worked with microglia to inhibit the development of the disease (Su et al., 2023). Its function in inhibiting disease progression is directly related to C–X–C motif chemokine receptor 6 (CXCR6), which is expressed on the surface of CD8⁺ T cells. When the gene is knocked out, the symptoms of the disease are more severe (Su et al., 2023; **Figure 5**).

Tregs

Tregs are a subset of CD4^+^ T cells that exhibit a high level of the transcription factor forkhead box protein P3 (Foxp3) and the CD25. Tregs can inhibit the pro-inflammatory immune response of other Th subsets, maintain immune homeostasis, and prevent the activation of autoreactive lymphocytes, thereby protecting the body from self-attack (Sojka et al., 2008), playing a vital part in immune regulation *in vivo*. The absence of Tregs in mice has been shown to trigger inflammation and fatal autoimmune diseases (Kim et al., 2007). According to existing evidence, impairment of the suppressive function related to Tregs is associated with the severity of AD (Liston et al., 2022). This is evidenced by a significantly reduced proportion of Tregs in the peripheral blood of patients (Ciccocioppo et al., 2019) and impaired cell suppressive function (Rosenkranz et al., 2007; Faridar et al., 2020), suggesting that Tregs can be modulated to provide therapies for NDDs (Faridar et al., 2020). In addition, Tregs may be able to prevent immune cells from entering the CNS, thereby reducing immune cell recruitment (Dansokho et al., 2016; **Figure 5**).

However, the effect of Tregs on AD is not one-sided; some experiments have found Tregs to be protective (Baek et al., 2016; Alves et al., 2017), while others have found Tregs to be detrimental (Baruch et al., 2015). Transient depletion of Foxp3⁺ regulatory T cells enhances immune cell recruitment to the brain, resulting in amyloid plaque clearance, reduced neuroinflammation, and improved cognition in AD models (Baruch et al., 2015). However, there is insufficient evidence to distinguish between the impacts of brain residency and peripheral Tregs.

• B cells

B cells, a type of antibody-producing lymphocyte, have been less well studied in research on AD. The number of B cells found in the blood of AD patients is significantly lower than the control group, and single-cell RNA sequencing of peripheral blood mononuclear cells shows a significant negative correlation between the patients’ clinical dementia rating score and the decline in B cells (Xiong et al., 2021). The depletion of B cells in AD mice showed a decrease in cognitive performance and an increase in Aβ deposits (Xiong et al., 2021). These findings suggest a potential involvement of B cells in AD.

**Figure 6:**
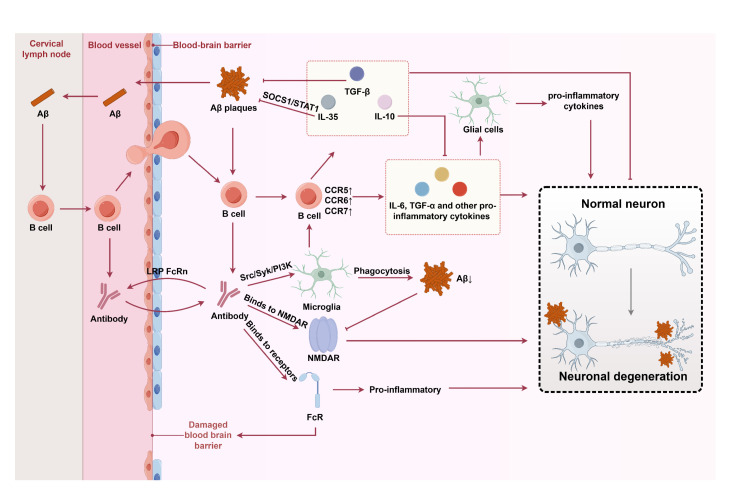
A schematic illustration depicting the involvement of B cells in AD pathogenesis. B cells can cross the blood-brain barrier, especially in pathological cases. Through FcRn and LRP, antibodies secreted by B cells enter the brain, where they promote Aβ clearance via Fc receptor-mediated phagocytosis by microglia. But in AD, the role of antibodies is dual, since their interaction with Fc receptors and NMDAR may exacerbate the disease. Meanwhile, B cells secrete cytokines (e.g., IL-10, IL-35, and TGF-β) and interact with glial cells. Created with Figdraw (www.figdraw.com, ID: UITTO01f90). AD: Alzheimer’s disease; Aβ: amyloid-beta; CCR: C-C chemokine receptor type; FcR: Fc receptor; FcRn: neonatal Fc receptor; IL: interleukin; LRP: low-density lipoprotein receptor-related protein; NMDAR: N-methyl-D-aspartic acid receptor; SOCS1/STAT1: suppressor of cytokine signaling 1/signal transducer and activator of transcription 1; Src/Syk/PI3K: proto-oncogene tyrosine-protein kinase Src/spleen tyrosine kinase/phosphoinositide 3-kinase; TGF: transforming growth factor.

B cells can act in AD pathology by secreting antibodies. Firstly, Aβ travels through the bloodstream or the lymphatic system to the cervical lymph nodes, where it is processed and presented by APCs and subsequently activates lymphocytes. Subsequently, these activated lymphocytes enter the bloodstream to secrete relevant substances and infiltrate brain tissue. In animal experiments, it was found that only a small fraction of antibodies in healthy mice could cross the BBB (Banks et al., 2002), whereas an increase in antibodies crossing the BBB was found in AD model mice, perhaps due to inflammation-induced damage to the BBB. Some of the antibodies they secrete can penetrate the BBB and act in the brain. But the role of antibodies is dual. On one hand, one study demonstrated that the non-amyloid-reactive antibody IgG improved microglia’s ability to bind and absorb Aβ, while more Aβ plaque deposition was found in 5×FAD mice lacking B cells (Marsh et al., 2016). On the other hand, once in the brain parenchyma, antibodies can cause damage to the brain, e.g., antibodies to synaptic N-methyl-D-aspartic acid receptor exacerbate symptoms in patients with progressive cognitive dysfunction (Pruess et al., 2012). Furthermore, AD, systemic inflammation, and aging, upregulate Fc receptors in perivascular macrophages and microglia in the CNS. The binding of Fc receptors to antibodies can result in neurodegeneration through the induction of inflammation (Peress et al., 1993; Fuller et al., 2014; **Figure 6**).

B cells can also regulate the progression of the disease through cytokine signaling. B cells secrete anti-inflammatory cytokines to limit the development of neuroinflammation, including IL-10, IL-35, and TGF-β. IL-10 alleviated Aβ_1–42_-induced learning and memory decline in rat models, and reduced Aβ_1–42_-induced increase in APP protein expression (Hou et al., 2013). IL-10 exerts its anti-inflammatory effects in the CNS mainly by inhibiting the expression and activation of inflammatory factors and their receptors (Wan-Qi et al., 2013). IL-35 from B cells inhibited beta-secretase 1 (BACE1) expression via the suppressor of cytokine signaling 1/signal transducer and activator of transcription 1 (SOCS1/STAT1) pathway, thereby reducing Aβ production in the brains of 5×FAD mice (Feng et al., 2023).

Pro-inflammatory cytokines (e.g., IL-6 and TNF-α) released by B cells activate glial cells and related pathways (e.g., NF-κB), leading to the release of more inflammatory cytokines and generating a cascade of downstream responses that worsen the condition (**Figure 6**).

• Natural killer cells

As an essential immune cell, NK cell belongs to the group of large granular lymphocytes responsible for killing abnormal cells in the body, such as senescent cells, viral infections and tumors, and in some cases involved in hypersensitivity reactions and autoimmune diseases. Combined with aging factors, there is evidence that the association between NK cells and AD may be related to chromodomain helicase DNA binding protein 6 (*CHD6*) (Li et al., 2024a). However, the study of NK cells is limited and controversial. Circulating NK cells have cytotoxic effects in AD (Rodriguez-Mogeda et al., 2024). In the experiment (Zhang et al., 2020b), which used an anti-NK1.1 antibody to deplete NK cells, it was found that neurogenesis was enhanced, neuroinflammation was reduced, and cognitive impairment was improved in 3×Tg-AD mice. There was a negative correlation between the Mini-Mental State Examination (MMSE) score analysis results and NK cell viability. Recent clinical studies have reported that the NK cell-based therapy SNK01 improves cognitive function in patients with AD. It was found that patients had reduced pTau181, Aβ_42/40_, and neuroinflammation (Biotech, 2023). NK cells can be activated by a variety of cytokines, such as IL-12, IL-15, or IL-18. NK cells can also be activated by identified target cells, such as infected cells, stressed cells, or cancer cells (Rodriguez-Mogeda et al., 2024). NK cells produce IFN-γ when activated (Solerte et al., 1998), and IFN-γ has been implicated in the pathogenesis of AD (Belkhelfa et al., 2014). Some experiments have found that NK cells in AD patients secrete higher levels of TNF and IFN-γ, and their release in NK cells is negatively correlated with cognitive function in patients (Solerte et al., 2000). IFN-γ stimulates other immune cells, especially macrophages, causing inflammation in the brain (Prinz and Priller, 2017). In addition, NK cells expressing Toll-like receptor 2 (TLR2) can also recognize Aβ, possibly suggesting that NK cells can elicit an immune response against Aβ (Rodriguez-Mogeda et al., 2024).

#### Role of inflammatory pathways in Alzheimer’s disease

The pathological process of AD involves numerous inflammatory signaling pathways (**Table 2**). Among them, the NF-κB-related signaling pathway is crucial to the mechanism of AD because it is a pathway that is widely engaged in the inflammatory response (Sun et al., 2022). NF-κB pathway can be activated by pathogen-associated molecular patterns, damage-associated molecular patterns, pro-inflammatory cytokines, stress signaling, and environmental factors (Cheung et al., 2022; Morrison et al., 2023; Cicchinelli et al., 2024; Li et al., 2025). NF-κB signaling is activated through both the canonical and non-canonical pathways, with the former being a key feature in the development of AD. In the canonical NF-κB pathway, the p65/p50 dimer is typically bound to the inhibitor of κB-α (IκBα) and sequestered in the cytoplasm. When the cell is stimulated (e.g., by TNF-α or IL-1β), IκBα is degraded by the proteasome, releasing the p65/p50 dimer (Sivamaruthi et al., 2023). Subsequently, the NF-κB dimer enters the nucleus and regulates the expression of genes, including *APP*, which are related to synaptic function, memory, and Aβ metabolism (e.g., *BACE1*), thereby regulating their expression (Sun et al., 2022). The Aβ produced by APP cleavage can in turn activate the NF-κB pathway, exacerbating amyloid metabolism dysregulation and deposition (Lopez-Rodriguez et al., 2021; Li et al., 2022a, c; Jung et al., 2023). Tau protein also participates in the activation of the NF-κB pathway in microglia. By genetically activating or inactivating NF-κB, researchers found that the NF-κB pathway enhances the ability of microglia to process tau and promotes the propagation of tau *in vivo* (Wang et al., 2022a). Another group of researchers found that the tau promoter contains a potential NF-κB binding site, suggesting a bidirectional regulatory relationship between inflammation and tauopathy (Dutta et al., 2023). Extracellular tau may activate tau transcription through TLR2/myeloid differentiation primary response 88/nuclear factor kappa-B (TLR2/MyD88/NF-κB), thereby forming a vicious cycle (Dutta et al., 2023). In addition, ApoE is another important factor in the pathogenesis of AD, and there is also a bidirectional regulatory relationship between NF-κB and ApoE. On one hand, in astrocytes, the upregulation of ApoE expression induced by Aβ is mediated by NF-κB (Du et al., 2005). When cells are stimulated with Aβ, the NF-κB pathway is activated, leading to a significant increase in ApoE expression. While cells pretreated with NF-κB-targeting double-stranded DNA decoy oligonucleotides showed reduced responsiveness to Aβ stimulation (Du et al., 2005). On the other hand, ApoE (e.g., ApoE4) can regulate NF-κB activity by downregulating Transgelin 3 expression, thereby regulating the inflammatory phenotype of astrocytes (Arnaud et al., 2022). The non-canonical NF-κB pathway is activated through members of the TNF receptor superfamily, which in turn activates NF-κB-inducing kinase. NF-κB-inducing kinase phosphorylates inhibitor of kappa B kinase to produce p52. p52/RelB dimers are then generated and enter the nucleus, where they regulate immune responses, especially in lymphoid organ development (Sun et al., 2022).

**Table 2:** Role of inflammatory pathways in Alzheimer’s disease

Signaling pathway	Function	Reference
NF-κB signaling pathway	Regulates neuroinflammation, activation of microglia, oxidative stress response, apoptosis, Aβ and tau deposition, and synaptic function.	Jha et al., 2019b; Yang et al., 2021; Sun et al., 2022; Sivamaruthi et al., 2023; Hu et al., 2024b; Zhao et al., 2024
MAPK signaling pathway	Regulates neuroinflammation, activation of microglia, mitochondrial dysfunction, apoptosis, Aβ and tau deposition, and synaptic function.	Lee and Kim, 2017; Luo et al., 2022
mTOR signaling pathway	Regulates neuroinflammation, tau phosphorylation, cerebral microvascular constriction, autophagy, and aggregation of Aβ and tau.	Cai et al., 2023; Kumari et al., 2023; Davoody et al., 2024
COX signaling pathway	Regulates neuroinflammation, affects Aβ clearance and tau aggregation, modulates tau phosphorylation, and synaptic plasticity.	Wang et al., 2017; Guan and Wang, 2019; Jung et al., 2019
PI3K signaling pathway	Regulates neuroinflammation, tau phosphorylation and Aβ deposition, synaptic plasticity, and oxidative stress response.	Pan et al., 2024; Bhatia et al., 2025

Aβ: Amyloid-beta; COX: cyclooxygenase; MAPK: mitogen-activated protein kinase; mTOR: mammalian target of rapamycin; NF-κB: nuclear factor kappa-B; PI3K: phosphoinositide 3-kinase.

Recent studies have also revealed that microRNAs, such as miR-25802 and miR-107-5p, participate in regulating NF-κB signaling pathway, leading to oxidative stress and the development of neuroinflammation (Hu et al., 2024b; Zhao et al., 2024). Moreover, NF-κB signaling plays a crucial role in Aβ clearance of glial cells. Activation of NF-κB not only encourages the production of amyloid proteins but also influences their clearance (Yang et al., 2021b). In the APP23 mouse model of AD, astrocytic NF-κB activation reduced amyloid plaque burden, likely through paracrine mechanisms and influencing APP processing (Yang et al., 2021b).

#### Interplay between the immunity cells, inflammation, and Parkinson’s disease

*Immune cells and neuroinflammation in Parkinson’s disease*

In 1988, McGeer et al. found HLA-DR^+^ reactive microglia in post-mortem tissue from PD patients, which was one of the first pieces of evidence connecting neuroinflammation to the pathophysiology of PD. Subsequently, microgliosis and astrogliosis were also observed in patients with PD or in animal models (Hou et al., 2017; Wilson et al., 2023). Microglia are crucial to the inflammatory process in PD. α-Syn can activate microglia through multiple mechanisms, including receptor-mediated signaling (Kim et al., 2013), phagocytosis (Xiong et al., 2025), and reactive oxygen species (ROS) (Rojo et al., 2014), leading to excessive microglial activation and inflammatory responses (Scheiblich et al., 2021b). The receptor-mediated activation of microglia is associated with several receptors, including TREM2 (Xiong et al., 2025), TLR (e.g., TLR2 and TLR4) (Scheiblich et al., 2021b), purinergic P2X7 receptor (P2X7R) (Jiang et al., 2015), RAGE (Long et al., 2022), and Lag3 (Mao et al., 2016; Deyell et al., 2023). TREM2 is involved in the phagocytosis of α-syn fibrils, and the degradation process generates truncated α-syn 1–103 protofibrils, which has strong seeding activity and promote the progression of PD (Xiong et al., 2025). The absence of TREM2 inhibits the spread and pathological progression of α-syn in the brain. After activation by TLRs, NLRP3 inflammasomes were activated in microglia, leading to the secretion of inflammasome-dependent inflammatory cytokines. This process is similar to that observed in AD (Scheiblich et al., 2021b). Increased NLRP3 expression causes caspase-1 activation, inflammatory cytokine release, and more ROS generation, all of which will further promote neuroinflammation and injury (Deyell et al., 2023). In this process, caspase-1 processes α-syn into a truncated, aggregation-prone form (Wang et al., 2016). However, activated microglia also contribute to the clearance of α-syn, which can be selectively degraded through TLR4-NF-κB-p62-mediated autophagy (Choi et al., 2020). By distributing through tunneling nanotubes, microglia jointly break down fibrillar α-syn cargo, reducing inflammation (Scheiblich et al., 2021a).

Astrocytes play a dual role in PD. In early PD, astrocytes play a major role in protecting against and limiting neuronal damage. They can alleviate disease progression by directly internalizing α-syn and releasing molecules that promote its degradation (Wang et al., 2023b). Ventral midbrain-derived astrocytes inhibit neuronal α-syn aggregation and transmission by regulating oxidative stress, mitochondrial stress, and the extracellular inflammatory environment in a paracrine manner (Yang et al., 2022). Chronic reactive astrocytosis can lead to the continuous production of reactive oxygen and nitrogen species, exacerbating neuroinflammation and amplifying neurotoxicity (Brash-Arias et al., 2024). On one hand, reactive microglia release various inflammatory cytokines (e.g., IL-1α), which cause astrocytes to switch to an inflammatory phenotype (McGeer et al., 1988). On the other hand, damage-associated molecular patterns (e.g., α-syn) can induce the activation of NLR family CARD domain containing 4 and NLRP3 inflammasomes in astrocytes, thereby inducing inflammation (Freeman et al., 2017). Aggregates form in the cells when α-syn accumulation beyond the ability of clearance in astrocytes, which encourages the production of pro-inflammatory cytokines (e.g., IL-1, IL-6, and TNF-α) and chemokines (e.g., CXCL1 and CX3CL1) (Lee et al., 2010; Wang et al., 2023b; Li et al., 2024b). The study found that the induction of pro-inflammatory cytokines and chemokines was correlated with the degree of α-syn accumulation in glial cells, and when α-syn is present in excess, it may transfer and accumulate between neurons and glial cells, thereby inducing the formation of pathological inclusion bodies and degenerative cellular changes (Lee et al., 2010). In addition, genetic abnormalities associated with PD can also cause changes in astrocyte function and neuroinflammatory levels. For example, GBA regulates astrocyte proliferation (Liu et al., 2012; Usenko et al., 2021; Cossu et al., 2023) and modulates the inflammatory response (Sanyal et al., 2020). Overexpression of the SNCA causes an increase in BBB permeability (Gu et al., 2010), which raises the possibility of peripheral immune infiltration.

Activated lymphocytes, apoptosis-prone lymphocytes, central memory T cells, Tregs, and B cells have all been found to be significantly increased in the peripheral blood of PD patients (Garfias et al., 2022). Besides, numerous infiltrating T cells and inflammatory cytokines have been discovered in the brain parenchyma and CSF of PD patients and *in vivo* models (Yoshimura et al., 2023; Sun et al., 2024), suggesting a close connection between peripheral immunity and PD. Stimulation by α-syn induces microglial overexpression of MHC-II and presentation of α-syn, subsequently triggering the activation and infiltration of CD4^+^ and CD8^+^ T cells (Harms et al., 2013; Williams et al., 2022). Although CD8^+^ T cells were the predominant type (Xu et al., 2023), CD4^+^ T cells seem to be more important: 1-methyl-4-phenyl-1,2,3,6-tetrahydropyridine (MPTP)-induced dopaminergic neuronal death is reduced in mice lacking CD4^+^ T cells, but there is no significant change in mice lacking CD8^+^ T cells (Garfias et al., 2022; Sun et al., 2024). There is an increase in Th1 cells, which correlates negatively with MMSE scores and positively with the Unified PD Rating Scale-I (UPDRS-I) scores (Yang et al., 2021a). Th1 cells encourage the production of pro-inflammatory cytokines such as TNF-α and IFN-γ (Moradi and Chapouly, 2023), resulting in neuroinflammation and damage. Th2 cells are decreased in PD patients and positively correlate with MMSE scores (Yang et al., 2021a). By stimulating astrocytes, the cytokines they generate (IL-4 and IL-13) can influence the maintenance and improvement of cognitive function (Sun et al., 2024). PD patients also exhibit elevated Th17 cell levels, which are positively correlated with UPDRS-I and UPDRS-IV scores, but negatively correlated with MMSE scores (Yang et al., 2021a). Th17 cells secrete IL-17, which may promote an immune response to clear pathological antigens, thus playing a role in immune protection to some extent. However, other findings suggest that Th17 cells may exacerbate DA neuronal damage in the substantia nigra by activating the NF-κB pathway (Sommer et al., 2018). The above results show a strong association between CD4^+^ T cell subtypes and cognitive, behavioural functions of PD patients.

**Figure 7:**
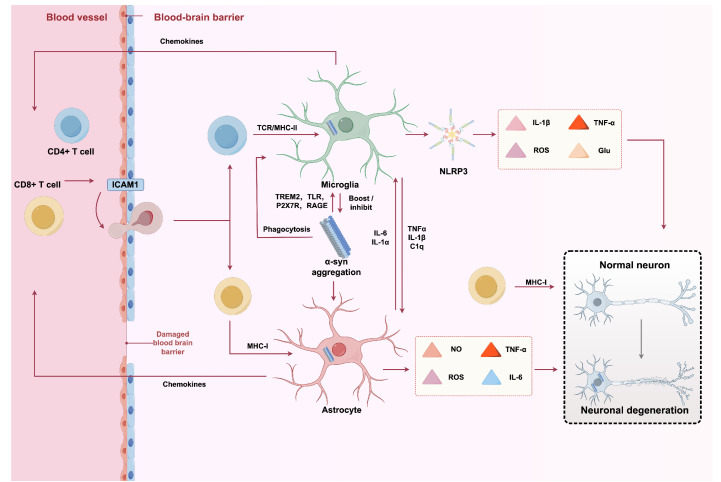
Cross-talk between T cells and central immune cells in PD. In PD, T cells cross the BBB, a process mediated by ICAM-1. α-syn interacts with microglia through TREM2, TLR, P2X7R, and RAGE, and T cells interact with microglia through TCR/MHC-II, leading to NLRP3 activation and release of pro-inflammatory cytokines such as IL-1β, TNF-α, and IL-6, which further promote neuroinflammation and damage. In astrocytes, α-syn accumulates and gradually forms intracellular aggregates, thereby inducing the release of pro-inflammatory cytokines. Besides microglia and astrocytes play dual roles in PD and can crosstalk via cytokines or the complement system. Created with Figdraw (www.figdraw.com, ID: OYTTIdac14). Glu: Glutamate; ICAM: intercellular adhesion molecule; IL: interleukin; MHC: major histocompatibility complex; NLRP3: NOD-like receptor family pyrin domain containing 3; P2X7R: purinergic P2X7 receptor; PD: Parkinson’s disease; RAGE: receptor for advanced glycation end products; ROS: reactive oxygen species; TCR: T cell receptor; TLR: Toll-like receptor; TNF: tumor necrosis factor; TREM2: triggering receptor expressed on myeloid cells 2; α-syn: α-synuclein.

Regarding B cells, the peripheral blood of PD patients showed a significant decrease in naive B cells and a significant increase in memory B cells (Wang et al., 2022b). Although B cell infiltration into the brain has not been observed in most rodent models, a few studies have reported B cell infiltration into the SN (Theodore et al., 2008). B cells can be activated by CD4^+^ T cells to produce antibodies, although their specific role in PD remains unclear. High-affinity antibodies may result from a T cell-dependent germinal center reaction following the binding of B cell receptors to their cognate antigens (Scott, 2022). In contrast, low-affinity antibodies are more likely to be generated through a T cell-independent process and are associated with B1 cells (Scott, 2022). This process leads to the production of polyreactive IgM. IgG can bind to Fc receptors present on glial cells and neurons, exerting downstream effects (Scott, 2022). In addition, animal studies have shown that α-syn expression induces a strong infiltration of pro-inflammatory CCR2^+^ peripheral monocytes into the SN (Harms et al., 2018). CD163, which is produced by monocytes/macrophages upon activation, is increased in the patients’ CSF (Nissen et al., 2021). However, the infiltration of peripheral mononuclear cells into the brain has not been clearly demonstrated in humans, and the findings in animal experiments still need to be further verified in humans. Besides, some evidence supported the potential act of neutrophils (Ji et al., 2008; Dobbs et al., 2016; Zhang et al., 2023b), macrophages (Chakraborty and Diwan, 2022; Schonhoff et al., 2023), NK cells (Dobbs et al., 2016; Menees and Lee, 2022; Holbrook et al., 2023), and dendritic cells (Mula et al., 2024) and PD (**Figure 7**).

*Role of inflammatory pathways in Parkinson’s disease*

One of the most significant and most commonly studied inflammatory pathways in PD is NF-κB related pathway (**Table 3**). Research has indicated that NF-κB activation is significantly increased in the brains of PD patients, particularly in the SN. Microglia play a central role in PD-related inflammatory signaling pathways, and upon stimulation by α-syn aggregates, they activate the NF-κB signaling pathway (Eo et al., 2024). A study using a 6-hydroxydopamine-induced rat model found that activated NF-κB upregulates p53, promoting apoptosis of DA neurons (Liang et al., 2007). Blocking NF-κB activation helps restore DA levels and reduce the degeneration of SN pars compacta (SNpc) DA neurons (Zhang et al., 2021). NF-κB mediates α-syn-induced microglial activation in PD, similar to its role in Aβ-driven AD inflammation. For example, in PD, the NF-κB signaling pathway promotes the release of pro-inflammatory cytokines and exacerbates neuronal damage by activating the NLRP3 inflammasome and an oxidative stress-related positive feedback loop (Singh et al., 2020; Eo et al., 2024). A study indicated that although NF-kB acts in PD and AD, pro-inflammatory pathways and their upstream transcription factors were not perturbed in PD, suggesting that inflammation may have less importance in late-stage PD compared to AD (Kelly et al., 2019). This may be because DA degradation is the main source of SN ROS, and as DA neurons are gradually lost, inflammation levels may decrease (Kelly et al., 2019).

**Table 3:** Role of inflammatory pathways in Parkinson’s disease

Signaling pathway	Function	Reference
NF-κB signaling pathway	Regulates neuroinflammation, microglial activation, oxidative stress response, apoptosis, synaptic function, and both the deposition and clearance of α-syn.	Singh et al., 2020; Wang et al., 2020b; Rocha et al., 2023; Eo et al., 2024
MAPK signaling pathway	Modulates neuronal stress responses, promotes inflammatory responses, and regulates aggregation and degradation of α-syn.	Wang et al., 2024b; Yokoya et al., 2025
mTOR signaling pathway	Regulates autophagy and inflammatory responses; may play neuroprotective or neurotoxic roles in PD; protects DA neurons from oxidative stress; promotes aggregate formation.	Khan et al., 2023; Ramasubbu and Devi Rajeswari, 2023; Liu et al., 2024a
COX signaling pathway	Promotes neuroinflammation and microglial activation, and accelerates α-syn accumulation.	Bartels and Leenders, 2010; Kumar et al., 2020
PI3K signaling pathway	Involved in cell proliferation and apoptosis, and contributes to the protection of dopaminergic neurons.	Wu et al., 2007

COX: Cyclooxygenase; MAPK: mitogen-activated protein kinase; mTOR: mammalian target of rapamycin; NF-κB: nuclear factor kappa-B; PI3K: phosphoinositide 3-kinase; α-syn: α-synuclein.

However, the NF-κB pathway may also alleviate PD. c-Rel activation is crucial for neuroprotection and anti-inflammatory control. Experiments using SH-SY5Y cells treated with 1-methyl-4-phenylpyridinium revealed that c-Rel activates the expression of anti-apoptotic genes, leading to upregulation of Bcl-xL, Bcl-2, and SOD2 protein levels (Wang et al., 2020b). Application of the c-Rel inhibitor exacerbates MPTP-induced damage to DA neurons, and mice lacking c-Rel develop late-onset Parkinsonian symptoms (Wang et al., 2020b). After the death of PD patients, researchers found that the DNA-binding activity of c-Rel was reduced in both SN and peripheral blood mononuclear cells, suggesting that c-Rel dysfunction may be one of the mechanisms underlying neuroinflammation dysregulation and DA neuron degeneration (Porrini et al., 2023). The clearance of α-syn depends on the activation of autophagy genes (e.g., p62) in microglia, which are regulated by NF-κB. Experiments on IKK2-NF-κB KO animals found an increase in the accumulation of misfolded α-syn in microglia (Rocha et al., 2023).

#### Interplay between the immunity cells, inflammation, and multiple sclerosis

*Immune cells and central nervous system injury in multiple sclerosis*

**Figure 8:**
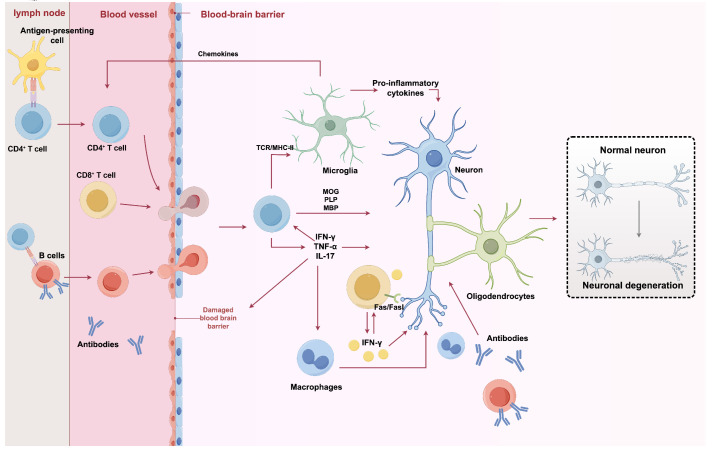
Cross-talk between immune cells and MS. In MS, abnormal immune activation leads to the infiltration of peripheral immune cells into the central nervous system across the blood-brain barrier, triggering neuroinflammation and demyelination. CD4^+^ T cells, activated by APCs, enter the CNS and release pro-inflammatory cytokines such as IFN-γ, TNF-α, and IL-17, causing oligodendrocyte damage and neuronal injury. CD8^+^ T cells contribute to MS pathogenesis through Fas/FasL and IFN-γ secretion. B cells are also involved in CNS infiltration and participate in the process of demyelination through cytokine secretion and production of antibodies against the central nervous system. Created with Figdraw (www.figdraw.com, ID: STRPTe1971). APCs: Antigen-presenting cells; Fas: Fas cell surface death receptor; Fasl: Fas ligand; IFN: interferon; IL: interleukin; MBP: myelin basic protein; MHC: major histocompatibility complex; MOG: myelin oligodendrocyte glycoprotein; MS: multiple sclerosis; PLP: proteolipid protein; TCR: T cell receptor; TNF: tumor necrosis factor.

MS is a typical autoimmune disease, and the key factor is considered to be an abnormality of the immune system. The characteristic pathological hallmark of MS is perivenular inflammatory lesions (Dobson and Giovannoni, 2019). Traditionally, the pathogenesis of MS has been thought to be primarily driven by T cells. However, an increasing amount of research has revealed that B cells, dendritic cells, and microglia are also crucial for the disease development. Despite this broader understanding, aberrant immune responses resulting from the infiltration of peripheral immune cells into the CNS remain the main driver of inflammatory disease activity in MS (Bar-Or and Li, 2021). The initiating factors of MS still need to be clarified by further research. The pathological process of the disease mainly includes two key steps: myelin destruction and inflammation (**Figure 8**).

The origin of MS lesions is thought to result from the infiltration of peripheral immune cells into the perivascular space of inflamed postcapillary venules (Tonietto et al., 2023). The inflammatory infiltrate in MS contains T cells, primarily MHC-I restricted CD8^+^ T cells (Dobson and Giovannoni, 2019). In the early MS, T cells can be found within the CNS lesions (Dendrou et al., 2015). Self-reactive T cells triggered by inflammation are transported from the peripheral blood to the brain in animal experiments, and they enter the CSF through the leptomeninges instead of the choroid plexus (Schläger et al., 2016). Autoreactive T cells targeting myelin antigens, such as Th1 and Th17 cells, are present in MS patients due to abnormal immune responses. Once activated, these cells can cross the BBB and secrete inflammatory cytokines (e.g., IFN-γ, TNF-α, and IL-17) in the CNS, leading to myelin and nerve damage. Th1 cells are considered the primary initiators and mediators of MS. They are present in the circulation and lymph nodes of patients and possess the ability to target myelin antigens such as myelin oligodendrocyte glycoprotein (MOG), proteolipid protein (PLP), and myelin basic protein (MBP) (Martinsen and Kursula, 2022). They trigger the activation of resident microglia and their differentiation into pro-inflammatory and neurotoxic phenotypes, causing neuroinflammation and neuronal damage. Additionally, IFN-γ and TNF-β derived from Th1 cells can activate macrophages, which in turn damage oligodendrocytes and contribute to pathological myelination (Liu et al., 2022b). It has also been found that astrocytes upregulate the expression of some chemokines (e.g., CCL20, CXCL10, and CXCL12) in response to IFN-γ, which further recruits Th1 and Th17. CCL2 also contributes to BBB disruption by promoting the disassembly and downregulation of tight junction proteins (Kunkl et al., 2022). This process further increases the infiltration of peripheral immune cells, exacerbates the immune response, and promotes disease progression. Th17, another type of T cell involved in MS, was found to increase in number during an attack (Liu et al., 2022b). IL-17 produced by Th17 cells increases the permeability of the BBB and migrates to the CNS through the expression of CCR6 (Melnikov and Lopatina, 2022).

Increased numbers of plasma cells and plasmablasts have been detected in the patients’ blood, and depletion of B cells using anti-CD20 antibodies has proven to be an effective treatment for MS (Wanleenuwat and Iwanowski, 2019; Seals et al., 2022; Kumar and Axtell, 2023). Early studies found infiltration of B cells and plasma cells. While plasma cells are mainly found in the meninges, infiltration of B cells is primarily observed in active lesions, meninges, and slowly expanding lesions (Frischer et al., 2009). These results suggest that B cells might have a role in the development of MS. B cells can also participate in the disease process through humoral immunity. The presence of CNS-targeting antibodies in the serum and CSF of MS patients suggests that humoral immune responses are involved in the inflammatory demyelination process, with targets including MOG (Sánchez-Vera et al., 2023). Some patients have elevated IgG (e.g., MOG) titers in their serum (Spadaro et al., 2016). Although the main research direction is IgG, IgM, and IgE also play a role (Seals et al., 2022; Sánchez-Vera et al., 2023). The presence of these B cells is closely associated with disease relapses in MS (Sánchez-Vera et al., 2023).

Demyelination is another key pathological step in MS. Inflammatory responses induce perivenular demyelination, which extends from the lesion edge into the surrounding normal-appearing white matter. In MS, damage to oligodendrocytes leads to myelin destruction, which induces blockage of electrical signal transmission and axonal transection (Dolati et al., 2017). In the early stages of MS, oligodendrocytes attempt to regenerate myelin; however, as the disease progresses, their repair ability declines, eventually resulting in increased neuronal damage and worsening of clinical symptoms (Tonietto et al., 2023). In addition, demyelination also promotes inflammatory activation processes, which further lead to BBB damage and stimulate the activation of immune cells and oxidative stress pathways (Dargahi et al., 2017). This positive feedback loop further drives disease progression.

*Role of inflammatory pathways in multiple sclerosis*

The mammalian target of rapamycin (mTOR) signaling pathway plays a critical role in the regulation of both innate and adaptive immune responses. mTOR can be divided into two complexes: mTOR complex 1, composed of mTOR, regulatory-associated protein of mTOR, mammalian lethal with SEC13 protein 8 (mLST8), and other associated proteins; and mTOR complex 2, composed of mTOR, rapamycin-insensitive companion of mTOR, mLST8, and additional components. Numerous signs, including growth hormones, glucose, and amino acids, can activate mTOR (Vakrakou et al., 2022). The phosphoinositide 3-kinase/protein kinase B (PI3K/AKT) pathway is the main upstream signaling pathway of mTOR and is involved in the regulation of apoptosis and inflammation. Activation of PI3K initiates a cascade of signaling events that lead to the phosphorylation and activation of mTOR, ultimately resulting in microglial activation (Thakur et al., 2023). mTOR ultimately increases NF-κB activity. Subsequently, microglia undergo a phenotypic change and produce neurotoxic effects. Overactivation of mTOR leads to BBB disruption, thereby promoting infiltration of peripheral immune cells (Zeng et al., 2022a).

The AKT/mTOR pathway is also one of the main signal cascades that control the development of oligodendrocyte precursor cells and myelin formation (Devanand et al., 2023), and prevent neuronal cell death and promote neural repair following injury (Li et al., 2022b). Researchers have observed significant involvement of the mTOR network in the early stages of oligodendrocyte maturation (Mammana et al., 2018). SC79, an activator of the Akt/mTOR signaling pathway, can inhibit abnormal apoptosis of oligodendrocyte precursor cells caused by excessive autophagy (Li et al., 2024e). Other studies have shown that activation of the protein kinase B/mammalian target of rapamycin/70-kDa ribosomal protein S6 kinase (AKT/mTOR/p70S6K) pathway enhances oligodendrocyte differentiation and myelin formation, and promotes the polarization of microglia M2 in rats (Ge and Li, 2023; Ge et al., 2024a), demonstrating a certain anti-inflammatory potential (**Table 4**).

**Table 4:** Role of inflammatory pathways in multiple sclerosis

Signaling pathway	Function	Reference
mTOR signaling pathway	Positively regulates myelination and remyelination; improves axonal conduction; essential for OPC development; regulates autophagy and apoptosis.	Vakrakou et al., 2022; Devanand et al., 2023; Ge and Li, 2023; Ge et al., 2024a; Li et al., 2024e; Man et al., 2024
Notch signaling pathway	Coordinates T cell differentiation and inflammation; regulates OPC proliferation, differentiation, and maturation; modulates myelin formation.	Juryńczyk and Selmaj, 2010; Devanand et al., 2023; Mora and Chapouly, 2023
Wnt signaling pathway	Regulates myelination and remyelination; restores blood–brain barrier integrity; limits immune cell infiltration.	Lengfeld et al., 2017; Huang et al., 2024
MAPK signaling pathway	Knockout or inhibition of p38γ promotes myelin repair and OPC differentiation; moderate activation upregulates myelin genes; overactivation leads to demyelination and axonal degeneration.	Ishii et al., 2016; Marziali et al., 2024
Nrf2 signaling pathway	Reduces oxidative stress; provides antioxidant effects; lowers inflammation levels.	Aliyu et al., 2024; Stys et al., 2024

MAPK: Mitogen-activated protein kinase; mTOR: mammalian target of rapamycin; Nrf2: nuclear factor erythroid 2-related factor 2; OPC: oligodendrocyte precursor cell.

#### Interplay between the immunity cells, inflammation, and amyotrophic lateral sclerosis

*The role of immune cells in amyotrophic lateral sclerosis*

The main pathological features of ALS include the loss of MNs and interneurons in the cortex and spinal cord, with the proliferation of microglia and astrocytes (McCauley and Baloh, 2019). Abnormal aggregation of TAR DNA-binding protein-43 (TDP-43) in MNs and glial cells is considered one of the pathogenic mechanisms of ALS. Through synaptic transmission, it can spread from the cortex to other parts of the brain and spinal cord (Liao et al., 2022). TDP-43 has both dose-dependent and age-dependent effects (Liao et al., 2022). TDP-43 is mostly found in the nucleus under normal conditions and is an important RNA-binding protein in the nucleus. It mainly functions by shuttling between the nucleus and cytoplasm. However, under pathological conditions, TDP-43 is cleaved in the cytoplasm, and the resulting nuclear localization signal-deficient fragments cannot return to the nucleus, leading to nuclear depletion of TDP-43 and pathological aggregates in the cytoplasm (Liao et al., 2022).

Astrocytes and microglia are thought to act in the non-cell autonomous processes of ALS (Stoklund Dittlau and Van Den Bosch, 2023). Single-cell sequencing revealed that astrocytes, oligodendrocytes, and microglia in ALS models exhibit a large number of differentially expressed genes (DEGs), with astrocytes (4.72% of expressed genes), oligodendrocytes (3.73%), and microglia (1.63%) showing the most significant changes (Liu et al., 2020). Another single-cell sequencing analysis of ALS patients similarly found that DEGs were highly enriched in non-neuronal cells, particularly glial cells, and their related functional pathways were mainly involved in energy metabolism (Shen et al., 2024). In microglia from the primary motor cortex of patients, genes such as *SOD1* and *TUBA4A* were significantly upregulated, suggesting potential pathogenesis of ALS (Shen et al., 2024). In the early stages of ALS, microglia primarily exhibit a protective M2 phenotype. An increase in ionized calcium binding adaptor molecule 1-positive (IBA1^+^) and CD68^+^ microglia has been observed near corticospinal MNs, indicating significant microglial activation (Brettschneider et al., 2012). This is consistent with the most common pathological regions in clinical cases. Microglia are involved in the clearance of pathological TDP-43, a process mediated by TREM2. When TREM2 is defective in mice, it reduces the clearance of TDP-43 by microglia and exacerbates the behavioral defects and neurodegeneration induced by human TDP-43 (Xie et al., 2022). In rNLS8 mice, human TDP-43 pathology can be reversibly induced in neurons. When PLX3397 was used during the early recovery stage of rNLS8 mice, the mice did not fully recover motor function, showing the protective role of microglia in early ALS (Spiller et al., 2018). In ALS models, IBA1^+^ rod-shaped microglia are significantly increased in the spinal cord. Some of these microglia are immunopositive for insulin-like growth factor 2 and leukemia inhibitory factor, suggesting they may secrete these neurotrophic molecules to exert neuroprotective or reparative effects (Kihira et al., 2007; Wiens et al., 2024). As the disease progresses, microglia shift from an M2 to an M1 phenotype, which is neurotoxic (Mimic et al., 2023). This polarisation can be caused by cross-talk between T cells and microglia. TDP-43 can activate microglia via CD14, triggering the NF-κB pathway and NLRP3 inflammasomes (Zhao et al., 2015). The experiment also found that the survival rate of TDP-25 A315T-treated MNs did not significantly change in the absence of microglia (Zhao et al., 2015). However, the same amount of TDP-25 A315T causes a considerable loss of MNs when microglia are present (Zhao et al., 2015). In other words, TDP-43 is not directly toxic to MNs, but rather exerts neurotoxicity through pro-inflammatory microglia (Zhao et al., 2015).

Astrocytic responses to ALS involve three major pathological events: inflammation, protein aggregation, and atrophy or cell death. In ALS, pro-inflammatory cytokines such as TNF-α, IL-1β, and IL-6 are secreted by activated astrocytes, which enhance the inflammatory response. The NF-κB pathway is involved in astrocyte pathology and promotes the production of inflammatory cytokines (Liao et al., 2022). The accumulation of TDP-43 and other proteins (e.g., FUS) in astrocytes disrupts their normal functions, including glutamate uptake and potassium buffering (Ferrer, 2017; Carey and Guo, 2022). *In vivo* models and ALS patients, dysfunction of excitatory amino acid transporter 2 leads to reduced uptake of glutamate by astrocytes, which causes excessive neuronal discharge, abnormal calcium influx in neurons and ultimately excitotoxicity (Mimic et al., 2023; Stoklund Dittlau et al., 2023). The misfolded proteins accumulate in astrocytes and also induce autophagy and endoplasmic reticulum stress, further impairing the function of astrocytes (Hu et al., 2019; Chua et al., 2022; Zanini et al., 2022).

Previous studies have reported the presence of T cell infiltration in the CNS of patients with ALS (Engelhardt et al., 1993; Motataianu et al., 2020). A low patient survival rate is linked to a high frequency of CD4^+^FOXP3^–^ Teff in the blood and CSF, indicating its potential to be a predictor of disease (Yazdani et al., 2022). T cells play a crucial role in coordinating and regulating the immune response in ALS. Th2 has neuroprotective properties, while the Th1 and Th17 subsets exhibit pro-inflammatory properties. In the early ALS, Th2 cells increase the release of anti-inflammatory cytokines and neurotrophic factors have a negative correlation with the development of disease, including TGF-β, brain-derived neurotrophic factor, and glial cell line–derived neurotrophic factor (Mimic et al., 2023). In the later stages of ALS, Th1 cells play a major role in promoting rapid disease progression, and eliminating Th1 cells may be an effective method for extending survival (Shao et al., 2013). CD8^+^ T cells interact with MNs via MHC-I molecules, directly inducing cytotoxic injury to MNs and secreting IFN-γ (Kaur et al., 2022). In mouse models, CD8^+^ T cells infiltrate the CNS and exacerbate MN loss during the symptomatic phase. Elimination of CD8^+^ T cells reduces spinal MN damage (Coque et al., 2019). B cells influence the pathological progression of the nervous system by producing antibodies and immunological factors. Multiple autoantibodies have been detected in the serum of patients, including antibodies to monosialotetrahexosylganglioside, Fas, and calcium channels (Sathasivam et al., 2001; Kollewe et al., 2015; Mimic et al., 2023). Studies suggest that these antibodies may cause neuronal injury by interacting with neurons and their membrane receptors.

Besides, altered serum antibody levels have been observed in ALS patients, including increased levels of IgG and IgM, with disease severity correlating with higher antibody levels (May et al., 2014). However, the results of antibody levels vary across the literature, perhaps as a result of variations in patient selection criteria (Mimic et al., 2023). It can be inferred that the alteration of humoral immunity is clear evidence that B cells play an important part in the pathology of ALS. Inflammatory cells of peripheral origin have been observed in the damaged areas of ALS, including mast cells (Engelhardt et al., 1993), monocytes (Engelhardt et al., 1993; Henkel et al., 2004; Butovsky et al., 2012), and NK cells (Hertwig et al., 2016), which may be related to the mechanism of the disease.

*Role of inflammatory pathways in amyotrophic lateral sclerosis*

STAT3 signaling is one of the fundamental pathways involved in the pathogenesis of ALS (Richardson et al., 2023; **Table 5**). In ALS patients and animal models, phosphorylated STAT3 accumulates in microglia, and this activation is often accompanied by increased neuronal damage (Shibata et al., 2009). STAT3 activation occurs mainly in glial cells of the spinal cord, especially in animal models (e.g., the SOD1G93A mouse model) (Ohgomori et al., 2017). STAT3 is involved in pathological processes such as TDP-43 protein aggregation, mitochondrial dysfunction, denervation of skeletal muscle, and excitotoxicity in ALS (Kato and Sakamoto, 2021; Zuo et al., 2021; Richardson et al., 2023). The promotion of TDP-43 protein aggregation by STAT3 may be related to oxidative stress and neuroinflammation (Zuo et al., 2021). The NLRP3 inflammasome may be directly regulated by STAT3 signaling, and its activation leads to the production of large amounts of pro-inflammatory cytokines (Zhu et al., 2021). The STAT3 signaling pathway also regulates the myogenic capacity of satellite cells when muscle is damaged and degenerated. However, in the case of ALS models, the conclusion that STAT3 activation promotes muscle regeneration does not apply (Tortarolo et al., 2024). In addition, the activation of STAT3 is critical for the formation of glial scars by reactive astrocytes after neurodegeneration, and the formation of glial scars can inhibit the repair of neurons and the regeneration of axons (Okada et al., 2009; Tortarolo et al., 2024).

**Table 5:** Role of inflammatory pathways in amyotrophic lateral sclerosis

Signaling pathway	Function	Reference
JAK/STAT signaling pathway	Regulates mitochondrial dysfunction, TAR DNA-binding protein-43 protein aggregation, neuroinflammation, excitotoxicity, and the myogenic capacity of satellite cells following muscle injury and degeneration.	Ohgomori et al., 2017; Kato and Sakamoto, 2021; Richardson et al., 2023
MAPK signaling pathway	Regulates inflammatory responses, alleviates mitochondrial damage-induced skeletal muscle atrophy, and regulates myogenesis.	Endo, 2023; Guan et al., 2025
mTOR signaling pathway	Activation leads to reduced autophagy, increased astrocyte reactivity, and regulation of cell proliferation and motor neuron toxicity.	Granatiero et al., 2021
Nrf2 signaling pathway	Regulates ferroptosis, alleviates oxidative stress; prevents oxidative stress and cell death induced by mutant superoxide dismutase 1 protein.	Sivandzade et al., 2019; George et al., 2022; Xiang et al., 2024
Notch signaling pathway	Regulates cell growth, synaptic plasticity, and neuronal survival; loss-of-function of the Notch gene leads to neuronal dysfunction and long-term spatial memory deficits.	Wang et al., 2015; Jha et al., 2022

JAK/STAT: Janus kinase/signal transducer and activator of transcription; MAPK: mitogen-activated protein kinase; mTOR: mammalian target of rapamycin.

#### Other neurodegenerative diseases

Other NDDs mainly include multiple system atrophy (MSA), and Huntington’s disease (HD), which are less common than the four mentioned above and have been studied less.

MSA is characterized by neuronal loss and glial proliferation in multiple regions in the CNS, with aggregated α-syn (Poewe et al., 2022). Unlike PD, in which aggregated α-syn forms eosinophilic inclusions called Lewy bodies primarily in DA neurons of the SNpc, MSA is characterized by filamentous α-syn accumulation in oligodendrocytes, forming glial cytoplasmic inclusions (Sian-Hulsmann and Riederer, 2024). Additionally, the α-syn pathology in the two diseases differs in both structural and biochemical characteristics. The initial stimulus in MSA remains unknown, but significant neuroinflammation and immune cell involvement have been demonstrated. Autopsy reports have revealed widespread microglial activation and diffuse T cell infiltration (Leńska-Mieciek et al., 2023). Significant microglial activation was found in cerebellar input, extrapyramidal and pyramidal motor structures. Moreover, microglial activation is increased in regions with a high α-syn burden (Gao et al., 2023). The pathogenesis of MSA is similar to that of PD. α-Syn can activate microglia through various receptors, including TLR4, mediating their phagocytosis of α-syn and the response process (Yan et al., 2023). However, α-syn aggregates can significantly reduce the phagocytic ability of microglia and trigger an inflammatory response. In addition, similar to PD, the activation of NLRP3 inflammasomes is also one of the potential pathological mechanisms, as described previously (Yan et al., 2023). MHC-presented α-syn can activate T cells, resulting in the differentiation of CD4^+^ T cells and the activation of CD8^+^ T cell, which releases cytokines that create a harmful inflammatory environment (Su and Zhou, 2021).

HD is an inherited NDD caused by a CAG repeat expansion in the huntingtin (*HTT*) gene (Saba et al., 2022). Mutant huntingtin inclusions are a key neuropathological hallmark of HD and can be detected before symptoms appear. Reactive microglia and astrocytes progressively accumulate in the HD brain throughout disease progression (Jiang et al., 2023). T cells may infiltrate the brain and be activated by elevated sCD27 in the CSF (Field et al., 2024). In HD, B cells, T cells, and NK cells all express mutant huntingtin, and this level of expression increases as the disease progresses (Field et al., 2024).

## Therapeutic Strategies Targeting the Immune System in Neurodegenerative Diseases

**Figure 9:**
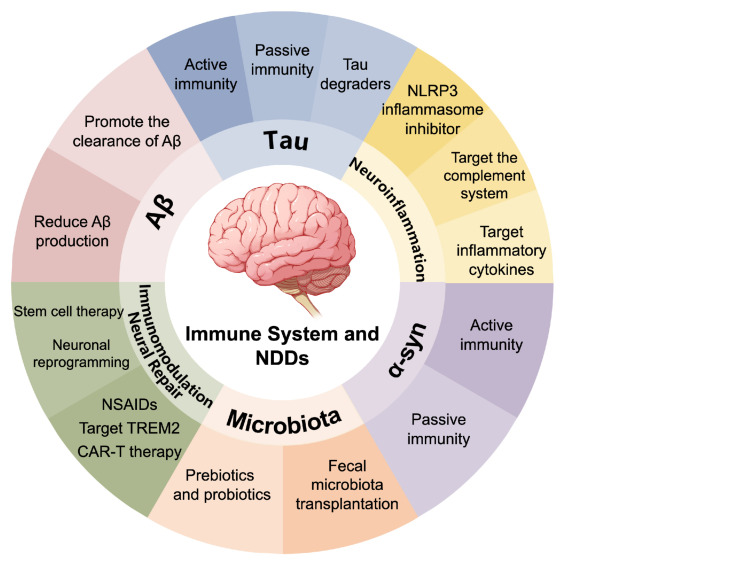
Schematic diagram of treatment strategies for NDDs. Common immunotherapeutic strategies for NDDs include targeting Aβ, tau, neuroinflammation, α-syn, the microbiota, immune modulation, and neural repair. These approaches mainly involve active immunization (e.g., using vaccines), passive immunization (e.g., antibody therapy), anti-inflammatory treatments, microbiota regulation, etc. Created with Figdraw (www.figdraw.com, ID: WPWYO8858b). Aβ: Amyloid-beta; CAR-T: chimeric antigen receptor; NDDs: neurodegenerative diseases; NLRP3: NOD-like receptor family pyrin domain containing 3; NSAIDs: nonsteroidal anti-inflammatory drugs; TREM2: triggering receptor expressed on myeloid cells 2; α-syn: α-synuclein.

The role of the immune system in NDDs has received increasing attention. Immune system-related therapies are emerging as a promising therapeutic strategy. This review will discuss common immune-related therapeutic strategies for NDDs, focusing on Aβ, tau, neuroinflammation, α-syn, the microbiota, immune modulation, and neural repair (**Figure 9**).

### Therapeutic strategies targeting amyloid-beta

AD and traumatic brain injury are NDDs associated with Aβ pathology. Therapeutic strategies targeting Aβ can be divided into two main aspects: reducing its production and enhancing its clearance.

Reducing Aβ synthesis in the brain can slow disease development and decrease amyloid accumulation. The generation of Aβ includes APP expression and APP cleavage. The main enzymes involved in the segmentation phase include β-secretase and γ-secretase (Conti Filho et al., 2023). By inhibiting their activity, the production of Aβ can be slowed down to a certain extent, thereby alleviating the symptoms of AD. However, although some drugs targeting β-secretase have achieved promising results in early clinical trials, they have failed in subsequent trials due to safety or efficacy issues (Hampel et al., 2021; Bazzari and Bazzari, 2022). Similarly, inhibitors targeting γ-secretase have been ineffective, and γ-secretase has been used as a target in phase 2 or phase 3 clinical trials, which have been unsuccessful (Yang et al., 2019). It was later found that these inhibitors inhibited the cleavage of Notch, another important substrate of γ-secretase, at the same time as they inhibited the cleavage of APP (Yang et al., 2019). Exploring cytokines with regulatory effects on related secretases may help treat AD. For example, IL-35 has been used to inhibit BACE1 expression through the SOCS1/STAT1 pathway, thereby reducing Aβ production (Feng et al., 2023).

In addition, Aβ clearance is currently one of the main strategies for the treatment of AD. Most patients have a relatively constant rate of Aβ production in the brain, and the efficiency of clearance is greatly reduced, resulting in Aβ accumulation (Su et al., 2021). Thus, impaired clearance of Aβ is thought to be a key factor in the deposition of Aβ in the brain and eventual plaque formation in AD patients (Su et al., 2021). Aβ clearance can be promoted in several ways. On one hand, the body’s immune system can be induced to produce more Aβ antibodies by active immunisation, i.e., by direct enhancement of the expression of the substance of interest, e.g., by injection of Aβ or fragments (e.g., AN1792 vaccine (Orgogozo et al., 2003), ACI-24 vaccine (Njavro et al., 2022)). On the other hand, it is also possible to supplement or augment the antibodies required to clear Aβ with passive immune responses, such as gantenerumab (Ostrowitzki et al., 2017), solanezumab (Honig et al., 2018), and aducanumab (Tolar et al., 2020), whereby the substance in question is injected directly into the patient from outside the body. However, passive immunity does not appear to be as effective (Alshamrani, 2023). There are also limitations to the removal of Aβ by antibodies, such as the presence of activated T-cell epitopes in the Aβ fragments, which, when seeded, can cause activation of T-cells and microglia, leading to severe adverse effects in patients (Lemere and Masliah, 2010). Coupled with the subsequent inflammatory response, this clearance process using antibodies can inadvertently impair the structural integrity of the cerebral vasculature, leading to BBB disruption, resulting in amyloid-related imaging abnormalities-edema and -hemorrhage (Kozberg et al., 2022; Kurz et al., 2022; Cozza et al., 2023).

**Figure 10:**
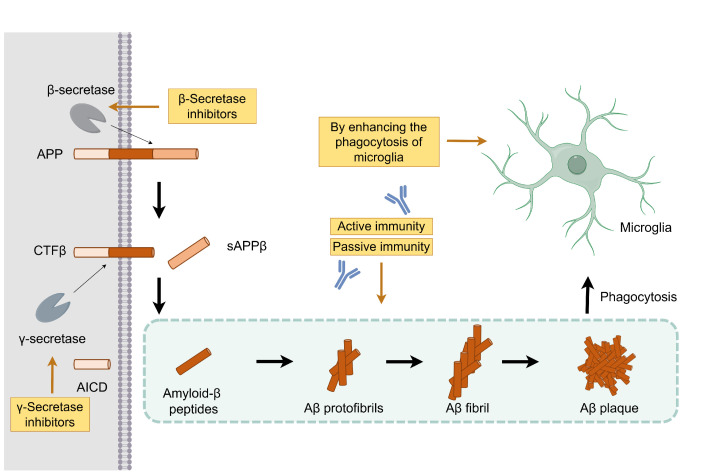
The main target of the therapeutic strategy against Aβ. In patients with AD, APP is cleaved by β-secretase to produce two fragments: CTFβ and sAPPβ. Subsequently, γ-secretase cleaves CTFβ to generate Aβ and AICD. The former (Aβ) gradually accumulates to form Aβ plaques, which progressively trigger neuroinflammation and neurodegeneration. Combined with the production process of Aβ, corresponding drugs can be developed from the above targets. Created with Figdraw (www.figdraw.com, ID: OTYTOe664e). AD: Alzheimer’s disease; AICD: amyloid precursor protein intracellular domain; Aβ: amyloid-beta; CTFβ: C-terminal fragment beta; sAPPβ: soluble amyloid precursor protein beta.

In addition, the clearance of Aβ can also be improved by directly enhancing the phagocytosis of microglia. It has been found that the phagocytosis of microglia is related to CD22, and the phagocytosis of mouse microglia can be significantly promoted by reducing CD22 or knocking down the related gene (Pluvinage et al., 2019). An experiment enhanced microglia phagocytosis by depolarization through optogenetic modulation strategies, successfully increasing the total amount of Aβ cleared and reducing amyloid deposition in the brain, and if intervened on this basis, complement C1q could simultaneously protect synapses from damage (Lv et al., 2024). This study demonstrates that controlling the phagocytic capacity of immune cells can regulate disease progression. On this basis, strategies for eliminating tau can be explored (**Figure 10**).

### Therapeutic strategies targeting tau

In AD and other tau-related NDDs (e.g., ALS), the overexpression, mis-localization and hyperphosphorylation of tau protein can lead to abnormal energy metabolism and behavioral or cognitive impairment. Phosphorylated tau competes with tubulin for binding to normal tau and other tubule-associated proteins, leading to microtubule depolymerisation and the formation of paired helical filaments within the neuron, which can impair neuronal structure and function (Wang et al., 2020a). Tau aggregation impedes the normal physiological functions of the endoplasmic reticulum and mitochondria, leading to oxidative stress, which in turn leads to tau aggregation, forming a vicious cycle (Ho et al., 2012; Pérez et al., 2018; Gorantla and Chinnathambi, 2021). Currently, there are several therapeutic strategies based on tau: active and passive immunity with tau vaccines, regulation of phosphorylation and aggregation inhibition, with the most promising therapeutic approaches including active tau vaccine active immunization and passive immunization with monoclonal antibodies (mAbs) (Wang et al., 2020a).

The goal of both strategies is to remove extracellular tau by antibody neutralisation of tau and to reduce the diffusion of tau proteins between cells. However, there are some side effects (Scheltens et al., 2021). Active immunization causes the body to respond with antibodies, such as AADvac1 and ACI-35/ACI-35.030 (Novak et al., 2019; Imbimbo et al., 2022; Chen and Yu, 2023). Safety, tolerability and immunogenicity of AADvac1 were favourable in phase 1 trials in mild to moderate AD, but poorly responded to in phase 2 trials (Novak et al., 2017; Chen and Yu, 2023). ACI-35.030 is a redesigned ACI-35 that has a stronger immune response than ACI-35 (Ji and Sigurdsson, 2021). Another type of immunotherapy is passive immunisation, in which anti-tau antibodies act against different tau epitopes. As of 2023, half of the 12 passive immune antibody drugs entering clinical trials have been terminated because of their insufficient clinical effectiveness against AD or progressive supranuclear palsy (Chen and Yu, 2023). Such drugs include RG7345 (Panza et al., 2016), UCB0107 (Vaswani and Olsen, 2020), and BIIB092 (Boxer et al., 2019).

In addition, new treatments include the use of selective tau degraders such as QC-01-175, which focus on clearing specific forms of tau in mutant neurons (Petrozziello et al., 2022; Silva et al., 2022). The principle is that the degrader molecule simultaneously recruits the target protein and an E3 ligase to form a ternary complex, which facilitates its ubiquitination and proteasomal degradation, thereby achieving targeted clearance (Silva et al., 2022). QC-01-175 specifically degrades pathological tau in a neuronal model derived from patients with FTD, and has no significant effect on normal tau in healthy neurons, showing good specificity (Silva et al., 2022). However, this therapy is currently limited to *in vitro* cell experiments and requires further research. HMPB-AAG nanomedicine is another agent that works by reducing p-tau levels. This formulation consists of the high-affinity heat shock protein 90 inhibitor 17-AAG and hollow mesoporous Prussian blue nanoparticles, representing a combinatorial therapeutic strategy. As a result, it promotes heat shock protein 40/heat shock protein 70-mediated p-tau degradation, reduces neuroinflammation, inhibits apoptosis, and tau-related pathology (Tang et al., 2022). However, it is important to note that drugs targeting mice may not be as effective in human patients (Wenger et al., 2023). Therefore, further study is required to build models that simulate a full disease cycle of humans, including tau ubiquitination and acetylation (Wenger et al., 2023).

### Therapeutic strategies targeting neuroinflammation

Neuroinflammation is a type of inflammation that occurs in the brain, and excessive activation of inflammatory molecules can cause brain damage. Neuroinflammation and microglial activation are central events in neurodegeneration. During the inflammatory process, glial cells release large amounts of pro-inflammatory cytokines, chemokines, and ROS, inducing infiltration of peripheral immune cells and neurodegeneration (Adamu et al., 2024). NDDs-related components that cause neuroinflammation include inflammasomes, complement system, and cytokines.

The NLRP3 inflammasome is considered a key contributor to neuroinflammation observed in NDDs such as PD. Pathological proteins including Aβ fibrils, α-syn fibrils, and TDP-43 can trigger NLRP3 inflammasomes (Zhao et al., 2015; Voet et al., 2019; Xiong et al., 2025). After the activation of the relevant receptor protein, the formation of the NLRP3 inflammasome involves the following steps: NLRP3 oligomerizes through interactions within its NACHT domain, recruits the adaptor apoptosis-associated speck-like protein containing a CARD (ASC) via Pyrin domain-Pyrin domain interactions, and the assembled ASC then recruits caspase-1, ultimately leading to the formation of the NLRP3 inflammasome (Kodi et al., 2024). NLRP3 activation increases the production of inflammatory cytokines and impairs pathological protein uptake by microglia. Activation of NLRP3 leads to the release of the pro-inflammatory cytokines IL-1β and IL-18 and gasdermin D-mediated pyroptosis (Han et al., 2023). The main treatment strategy targeting NLRP3 inflammasomes is to inhibit the above process using inhibitors. For example, inhibiting adenosine triphosphatase activity and blocking NLRP3 oligomerization (Shi et al., 2022b), disrupting the interaction of Pyrin domain-Pyrin domain (Coll et al., 2022), and specifically binding to NIMA-related kinase 7 (Kodi et al., 2024). Specific NLRP3 inflammasome inhibitors include MCC950, POPs, CY-09, OLT1177, etc., and non-specific inhibitors include BAY-117082, nonsteroidal anti-inflammatory drugs (NSAIDs), and Bruton’s tyrosine kinase (Kodi et al., 2024).

Complement in the brain is mainly derived from microglia and astrocytes, and when activated, mediates immune and inflammatory responses, primarily for promoting phagocytic clearance of pathogens and host harmful substances following injury and infection (Bohlson and Tenner, 2023). C3, CR1, C1q, and C3aR are implicated in Aβ metabolism. C4, C3, and CR1 play a role in tau pathology. C1q and CR1 are involved in neuroinflammation. C1q, C3, and CR1 are involved in synapse formation (Zhu et al., 2023b). C1q and C3d deposition was observed in ALS MNs, astrocytes, and microglia, and C1q and C4 overexpression in spinal cord microglia (Sta et al., 2011). Some drugs work by targeting the complement system. For example, C1q inhibitor ANX005 inhibits the function of the classical pathway and has been shown to have a beneficial effect in AD mouse models (Gagnon et al., 2023). Antibodies targeting C7 can block the formation of the membrane attack complex and have been shown to prevent synaptic loss, reduce neuroinflammation, and decrease amyloid burden in AD mouse models (Zelek et al., 2025). The complement system plays an essential part in the pathological genesis of NDDs, but its specific mechanism is still lacking in research. In addition, immunotherapies targeting cytokines have also been explored (Shetty et al., 2025). For example, inhibiting soluble TNF-α using small molecule inhibitors can significantly reduce neuroinflammation (Barnum et al., 2014). The use of anti-TNF-α mAbs effectively reduces Aβ plaque formation and tau phosphorylation (Chang et al., 2017).

### Therapeutic strategies targeting α-synuclein

**Figure 11:**
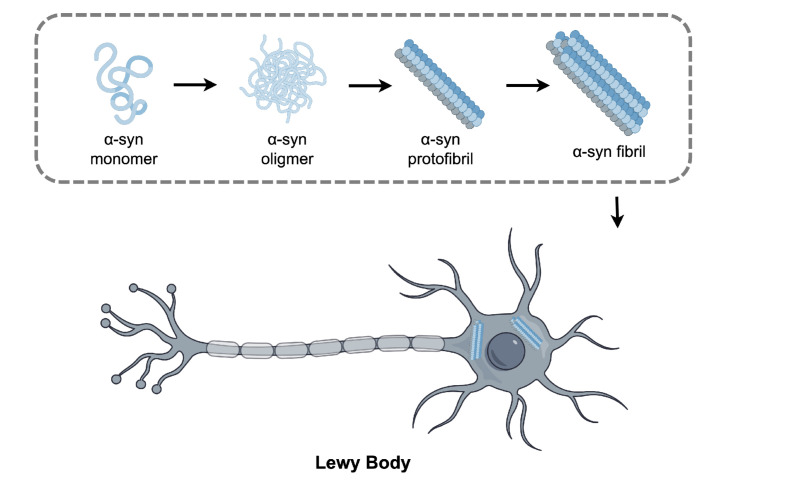
α-Syn exists in multiple protein conformations. α-Syn exists initially as monomers and progressively aggregates into oligomers, protofibrils, and fibrils. These fibrillar structures accumulate within the neuronal cell body, forming Lewy bodies, which is the hallmark of PD. Created with Figdraw (www.figdraw.com, ID: AOOWW81588). α-syn: α-Synuclein; PD: Parkinson’s disease.

α-Syn consists of three parts: an amino-terminal lipid-binding region, a central hydrophobic non-Aβ region, and a disordered carboxy-terminal. Due to its structural flexibility, α-syn can exist in multiple protein conformations. Under normal conditions, α-syn is a protein that is mainly located at presynaptic nerve terminals and is involved in synaptic function. It is involved in synaptic activity, vesicle trafficking, and the regulation of DA release (Dehay et al., 2015). During abnormal aggregation, it gradually becomes oligomers and fibrils, accumulates in cells, causes degeneration and death of DA neurons, and thus triggers PD (**Figure 11**). In summary, this physiological process can be divided into three main parts: synthesis, aggregation, and clearance. Common immune strategies currently include active immunization and passive immunization.

Active immunization can provide long-term effective clearance, avoid repeated dosing, and also has a certain preventive effect. However, its limitations lie in the unpredictable response of the body after induction (Orgogozo et al., 2003), especially in older adults. Active immunization is mainly intramuscular (Henríquez and Narayan, 2023). The main strategy of this approach is to construct vaccine with the same fragment as α-syn, or to use a full-length human recombinant α-syn for vaccination (Masliah et al., 2005; Park et al., 2023). This method reduces protein aggregation by inducing antibody production by B cells in a T cell-dependent manner and by promoting phagocytosis through the binding of antibodies to α-syn (Nimmo et al., 2023). The highest-affinity antibodies all target the C-terminus of α-syn (Nimmo et al., 2023). The effects of antibodies on α-syn include: (1) promoting the degradation of α-syn deposits, (2) inhibiting aggregation and toxicity, (3) preventing cellular uptake of α-syn, and (4) inhibiting its transmission and spread (Henríquez and Narayan, 2023). Such vaccines include Affitope PD01A/PD03A and UB-312, which have good specificity and do not activate T cells (Knecht et al., 2022).

Passive immunization directly uses antibodies that target different domains of α-syn. Although it requires repeated treatments, it can effectively control many parameters, such as binding affinity and antibody concentration. It is more flexible, allows rapid intervention when side effects occur, and avoids T cell activation (Knecht et al., 2022). In passive immunization, the drug is administered intravenously or subcutaneously, and the drug must cross the BBB to reach the brain parenchyma to exert its effect (Henríquez and Narayan, 2023). It mainly targets extracellular α-syn, so it is more effective in the aggregation and clearance stages. It can also effectively prevent the spread of α-syn and reduce its toxicity. Since antibodies targeting the C-terminus are more effective at clearing α-syn, drug development in this area has primarily focused on the C-terminal. Antibodies can also target membrane receptors on recipient cells, effectively reducing their response to and uptake of α-syn. For example, anti-TLR2 antibody therapy significantly reduces the accumulation of α-syn deposits in neurons and astrocytes, and alleviates inflammation by inhibiting downstream NF-κB (Kim et al., 2018). Although these methods can effectively reduce α-syn pathology, the risk of off-target effects should not be ignored (Chatterjee and Kordower, 2019).

### Therapeutic strategies targeting the microbiota

Our bodies contain microbes that are involved in a number of pathogenic and regulatory processes. Numerous mechanisms, including the immune system, neuroendocrine mediators, and transfer to the brain, allow microorganisms to influence nervous system activity (Dai et al., 2022). Among them, the gut microbiota is the main research object in NDD microbiology research.

Gut microbiota dysbiosis has been found in patients with NDDs such as AD, PD, MS, and ALS (Scheperjans et al., 2015; Bäuerl et al., 2018; Zhuang et al., 2018; Engelenburg et al., 2022; Bello-Corral et al., 2023; Ordoñez-Rodriguez et al., 2023; Proano et al., 2023; Zhang et al., 2023d, 2025; Chen et al., 2024; Zou et al., 2024). Compared with the cognitively normal control group, AD and PD patients had reduced gut microbiota diversity and significant changes in the composition. For example, *Akkermansia*, *Proteobacteria*, *Bifidobacterium*, and *Prevotella* were found to be more abundant in AD patients and PD patients (Aho et al., 2019; Hung et al., 2022). In MS, the abundance of *Bacteroides*, *Parabacteroides*, *Prevotella* decreased, while the abundance of *Blautia*, *Akkermansia* increased (Freedman et al., 2018). In ALS patients, a significant increase in Dorea was observed, while *Oscillibacter*, *Anaerostipes*, and *Lachnospiraceae* were significantly reduced (Fang et al., 2016).

The gut microbiota is essential for host development, digestion, behavior, and the immune system, while dysbiosis can also impact cognition and memory (Dai et al., 2022). The gut microbiota communicates with brain via a variety of pathways, collectively referred to as the microbiota-gut-brain axis (MGBA) (Morais et al., 2021; Zhou et al., 2024a). In short, gut microbiota is a regulatory factor in the progression of NDDs. For example, a significant correlation has been found between gut microbiota dysbiosis and the onset of NDDs (Mitrea et al., 2022; Zeng et al., 2023). Animal studies have shown that transplanting fecal microbiota from AD patients into microbiota-depleted young rats can induce cognitive deficits in hippocampal-neurogenesis dependent behaviors in models (Grabrucker et al., 2023). In another study using α-syn overexpression mouse models, microbiota depletion significantly alleviated neurological deficits compared to controls, while mice transplanted with fecal microbiota from PD patients exhibited significantly worsened motor symptoms (Sampson et al., 2016).

There are three main communication pathways between gut microbiota and brain: systemic humoral pathway, cellular immune pathway, and neural pathway (Agirman et al., 2021). The mechanism of the systemic humoral pathway is mainly that intestinal inflammation increases the release of systemic inflammatory factors (e.g., IFN-γ, IL-1β, IL-6, and TNF-α). The BBB will be damaged if inflammatory factors are not controlled. This allows toxins and pathogens to enter the brain parenchyma, activate local immune cells, and cause neuroinflammation. The cellular immune pathway mainly involves intestinal immune cells migrating to the brain and contributing to the development of neuroinflammation. The neural pathway, which is mainly related to the vagus nerve, senses microbiota metabolites such as short-chain fatty acids (SCFA) through afferent nerves, transmits information to the CNS, and triggers a series of responses (Bonaz et al., 2018). The vagus nerve can also indirectly sense signals from gut microbiota through enteroendocrine cells. Upon detecting stimuli from the microbiota metabolites, enteroendocrine cells can release serotonin 5-hydroxytryptamine, which activates 5-hydroxytryptamine type 3 receptors expressed on vagal afferent fibers, thereby transmitting signals to the CNS (Bonaz et al., 2018). By regulating and controlling related pathways, the development of NDDs may be effectively controlled.

Gut microbiota dysbiosis can be ameliorated by fecal microbiota transplantation, thereby alleviating NDDs (Ma et al., 2024). Fecal microbiota transplantation increases SCFA (e.g., butyrate), which regulates microglial maturation and function through free fatty acid receptor 2 signaling. In one experiment, it was found that microglia in the brains of germ-free mice exhibited significant differences in gene expression, many of which were related to microglia activation, phagocytosis, pathogen recognition, and other functions (Erny et al., 2015). In addition, the researchers found that the homeostatic status of microglia in germ-free mice was severely disrupted and exhibited an immature phenotype (Erny et al., 2015). However, after four weeks of SCFA mixture administration, microglia gradually returned to normal, while the microglia morphology of free fatty acid receptor 2 knockout mice showed abnormal morphology (Erny et al., 2015). Other reports have shown that butyrate can regulate the expression of the tyrosine hydroxylase (*TH*) gene through a cyclic adenosine monophosphate-dependent signaling pathway, thereby influencing DA synthesis (DeCastro et al., 2005). This reveals the connection between SCFA and PD from another perspective. SCFAs are also natural inhibitors of histone deacetylases (He et al., 2020). It is known that overexpression of histone deacetylases activates glycogen synthase kinase 3, thereby promoting excessive phosphorylation of tau and inhibiting its degradation. The use of histone deacetylase inhibitors effectively reduces tau phosphorylation in the hippocampus of model animals and restores their spatial memory function (Ricobaraza et al., 2009; Gupta et al., 2020). Besides, the functions of SCFA also include: (1) repairing leaky gut, (2) reducing inflammation, (3) regulating immune cells, (4) maintaining healthy mitochondrial function, and (5) inhibiting oxidative stress (Yadav et al., 2022). Fecal microbiota transplantation reshapes the gut microbiota to downregulate pro-inflammatory cytokines and inhibit the activation of inflammatory pathways such as the NLRP3 inflammasome and NF-κB signaling (Ma et al., 2024). Ultimately, it reduces the aggregation of pathological proteins α-syn and Aβ and alleviates NDDs (Ma et al., 2024).

In addition, prebiotics and probiotics are thought to alleviate NDDs by regulating gut microbiota. For example, *Lactobacillus plantarum* (*L.plantarum*) has been shown in AD models to enhance synaptic plasticity, restore intestinal barrier integrity, reduce Aβ deposition, and improve cognitive function (Hu et al., 2024a). In PD, *L.plantarum* has been shown to continuously improve motor deficits and protect DA neurons in the brain (Ma et al., 2023). In MS models, *L.plantarum* alleviated cuprizone-induced demyelination in rats (Chen et al., 2024). Other probiotics with therapeutic potential for NDDs include *Bifidobacterium breve* (Reiriz et al., 2025), *Lactobacillus rhamnosus* (Heydari et al., 2025), *Clostridium butyricum* (Stoeva et al., 2021), etc.

In human trials, although no significant improvement was observed in cognitive function assessments (e.g., AD Assessment Scale-Cognitive, MMSE, activities of daily living, and clinical dementia rating) after 12 weeks of probiotic treatment in AD patients, the treatment group showed a trend toward slowing the rate of symptom deterioration (Hsu et al., 2023). Probiotic treatment in cognitive impairment patients has been shown to effectively improve cognitive function and sleep quality (Fei et al., 2023). Although clinical trial data on the efficacy of probiotics is limited, probiotic supplements may serve as a potential treatment.

Not only the gut microbiota, but there has been evidence that the oral microbiota is associated with NDDs. The oral microbiota is the second largest microbial community in the human body after the intestinal microbiota (Tuganbaev et al., 2022). The oral microbiota, which is similar to the gut microbiota, can also contribute to systemic inflammation when dysbiosis occurs. However, unlike the gut microbiota, the oral microbiota is located in the head, closer to the brain, and the oral and maxillofacial region has facial nerves, trigeminal nerves, and abundant blood vessels. The oral microbiota and its virulence factors can enter the brain through blood vessels and nerves in certain disease conditions (Dai et al., 2025). Many studies have clarified the dysbiosis of the oral microbiota in NDDs and its correlation with NDDs (Li et al., 2022d; Kong et al., 2024; Rei et al., 2024; Wang et al., 2024a). For example, a case-control study found a correlation between chronic periodontitis in women and MS (Li et al., 2022d). An association has been observed between the severity of oral-motor deficits and oral dysbiosis in ALS patients; however, this phenomenon is present in bulbar-onset ALS patients but not in spinal-onset ALS patients (Kim et al., 2022). In animal studies, oral microbiota have been shown to exacerbate the progression of NDDs (Kim et al., 2022). A study showed the causal relationship in mouse models: oral infection with *Porphyromonas gingivalis* (*P. gingivalis*) led to brain colonization and subsequent brain Aβ production (Dominy et al., 2019). Another study showed that in MPTP-induced PD mice, ligature-induced periodontitis with subgingival plaque application worsened motor dysfunction, microglial activation, and DA neuron loss (Bai et al., 2023; Rei et al., 2024). And some researchers believe that the oral microbiota may influence the pathogenesis of AD by promoting oral barrier permeability, allowing the oral microbiota or inflammatory mediators to enter the bloodstream, or through the transfer of microorganisms from the oral cavity to the brain (Fox et al., 2019). The application of probiotics is one of the common strategies to maintain microbial homeostasis. Oral probiotics can competitively inhibit the nutrient intake of periodontal pathogens (Kong et al., 2024). This theory has already been applied in animal studies. Studies have shown that *P. gingivalis* infection can lead to memory impairment and the development of AD (Kazemi et al., 2024; Liu et al., 2024b; Huang et al., 202b). By blocking the adhesion and invasion of *P. gingivalis* in gingival epithelial cells, probiotic strains from the genera *Bifidobacterium* and *Lactobacillus* can alleviate the progression of the disease (Kazemi et al., 2024). It has been shown that *Lactobacillus* can significantly improve memory deficits and hippocampal damage in AD model (Kazemi et al., 2024). Furthermore, in animal models of AD, *Bifidobacterium breve* (*B. breve*) MCC1274 has shown potential in improving cognitive function (Abdelhamid et al., 2024, 2025). Mice with Aβ_1–40_ solution-induced working memory impairment benefit greatly from daily oral dose of *B. breve* (Abdelhamid et al., 2025). Another experiment conducted on App NL-G-F mice also found that intervention with *B. breve* MCC1274 improved cognitive dysfunction in aged mice. However, *B. breve* MCC1274 did not significantly affect Aβ_40_ and Aβ_42_ levels in mouse models, which may indicate that it acts through an amyloid-cascade-independent pathway (Abdelhamid et al., 2024). Although the therapy has therapeutic potential in animal models, it is used less in clinical trials.

### Immunomodulation and neural repair

#### Immunomodulation

Immunomodulation therapy refers to a treatment strategy that directly acts on immune cells or immune-related molecules to regulate immune responses (Duggan et al., 2025). Since NDDs are commonly accompanied by neuroinflammation and immune cell activation, interventions targeting the immune system are considered to have potential therapeutic value.

NSAIDs play a crucial role in the initial research on immunomodulation. These drugs are a class of non-opioid drugs with analgesic, antipyretic, and anti-inflammatory properties, primarily inhibiting COX activity and other regulatory enzymes. Compared to healthy individuals, patients with AD, PD, MS, and ALS exhibit elevated COX-2 levels in the CNS (Yiangou et al., 2006; Kumar et al., 2020; Shah et al., 2024), showing a potential relationship between COX-2 and the pathogenesis of NDDs (Shah et al., 2024). Epidemiological studies suggest that anti-inflammatory drugs may help prevent NDDs (e.g., AD and PD; Wahner et al., 2007; Ali et al., 2019), but the results in this area are controversial and conflicting (Bogár et al., 2022). Research has also found that NSAIDs only show a preventive effect when used long-term before Aβ begins to accumulate; once Aβ accumulation has occurred, such drugs not only have limited efficacy but may even worsen the condition, possibly because NSAIDs can inhibit already activated microglia (Imbimbo, 2009). Besides, randomized clinical trials of NSAIDs in AD patients have failed to show satisfactory results (Ali et al., 2019) and may even increase the risk of gastric ulcers and intestinal bleeding (Bogár et al., 2022). Therefore, the use of NSAIDs is currently not recommended (Duggan et al., 2025).

As mentioned above, the activation of microglia and the neuroinflammation they mediate are key factors in NDDs. Therefore, targeting microglia has also been considered a viable strategy for the treatment. As the main immune cells of the CNS, microglia play a protective role in NDDs by clearing pathological products, but they may also induce inflammatory damage due to excessive activation. TREM2 is a membrane receptor closely related to the microglia phagocytic function, mediating the clearance of Aβ, α-syn, and myelin debris (van Lengerich et al., 2023; Yin et al., 2024). TREM2 activation helps enhance microglial phagocytosis and alleviate neuroinflammation and cognitive behavior, while TREM2 deficiency impairs the ability of microglia to phagocytose Aβ fibrils/aggregates (Meilandt et al., 2020; Lin et al., 2024). In the PD model, TREM2 suppresses neuroinflammation and neurodegeneration by negatively regulating TNF receptor-associated factor 6/TLR4-mediated mitogen-activated protein kinase and NF-κB signaling pathway activation (Ren et al., 2018). TREM2 signaling depends on its ligands (e.g., lipid molecules, ApoE, and Aβ oligomers) binding to the receptor, which then recruits downstream pathways such as spleen tyrosine kinase via DNAX activating protein of 12 kDa (DAP12) to activate microglia (Guo et al., 2024). Currently, several therapeutic drugs targeting TREM2 are under development. For example, AL002 is a humanized IgG1 mAbs targeting the extracellular domain of human TREM2, which binds to TREM2 on the surface of microglia, promoting the formation and stabilization of the TREM2-DAP12 receptor complex (Guo et al., 2024; Ma et al., 2025). In the phase 1 study, the treatment group demonstrated a decrease in soluble TREM2 and an increase in soluble colony-stimulating factor 1 receptor in the CSF (Long et al., 2024). Soluble TREM2 (sTREM2) is a cleavage product of the TREM2 receptor and serves as a biomarker of enhanced microglial function, while soluble colony-stimulating factor 1 receptor is a marker for TREM2 signaling in CSF (Suárez-Calvet et al., 2016; Long et al., 2024). However, the primary endpoint measured by the sum of boxes of the clinical dementia rating was not met in the phase 2 study, and the secondary endpoint was also failed (Alector, 2024). Other drugs targeting TREM2 include the TREM2 antibody VHB937 and the small molecule agonist DNL919 (Stangel et al., 2024; Ashvin et al., 2025).

Cell-based immunotherapy is another approach to treat NDDs, such as chimeric antigen receptor (CAR)-T cell therapy and CAR-macrophage therapy. CAR-T is a type of adoptive immunotherapy that involves preparing T cells that specifically recognize target antigens *in vitro* and injecting them into the patient (Nakashima and Kagoya, 2024). CAR-T was originally used mainly for malignant tumors, but now people are beginning to explore applications outside this field, such as MS (Rankin and Shah, 2024). mAbs therapies targeting B cells have certain limitations in that they cannot adequately penetrate the CNS, allowing certain cells located therein to avoid depletion (Konitsioti et al., 2024). CAR-T therapy possesses tissue-penetrating capabilities, allowing it to enter the CNS and expand locally; however, it also carries the risk of neurotoxicity (Rankin and Shah, 2024). One report described two MS patients treated with CD19 CAR-T cells, providing the first evidence of early safety, pharmacokinetics, and target cell effects following CAR-T application in MS (Fischbach et al., 2024). In patient 1, a significant and rapid decrease in oligoclonal band counts was observed 14 days after infusion; and CAR-T cell enrichment in CSF was detected in both patients, accompanied by reduced disease progression and inflammatory activity, with no signs of early neurotoxicity (Fischbach et al., 2024). However, this therapy still requires large-scale and long-term follow-up validation. If this technology can integrate the targeted ability of innate immune cells against antigens, then theoretically, modified macrophages in the brain can efficiently recognize and degrade specific pathological proteins (Duggan et al., 2025). One study demonstrated that macrophages expressing a CAR targeting Aβ significantly absorbed Aβ and amyloid plaques both *in vitro* and *in vivo* (Kim et al., 2024a).

#### Neural repair

Because of the limited regenerative and repair capacities of the CNS, most NDDs focus on controlling their symptoms and slowing their progression. Therefore, neural repair strategies have been proposed to treat NDDs. This section primarily focuses on stem cell therapy and neuronal reprogramming.

Another name of stem cell therapy is regenerative therapy. The potential mechanisms of this therapy are complex and multifaceted, including neuronal replacement, paracrine, immunomodulation, and neurotrophic support (Deng et al., 2025). Currently, most stem cell-based studies remain at the preclinical stage and mainly in animal models (Song et al., 2024). Research results show that transplanted stem cells replenish or replace degenerated neurons by generating new neurons to restore the function of the CNS. For example, in PD, stem cells can differentiate into DA neurons and integrate into existing neural circuits to restore function (Deng et al., 2025). The mechanisms of stem cell therapy also include immunomodulation (Li et al., 2012), involving the regulation of cytokine release and the control of neuroinflammation (Sivandzade and Cucullo, 2021). The mechanism by which stem cell therapy promotes endogenous neurogenesis may be related to controlling oxidative stress level and promoting the expression of neurotrophic factors in newly generated cells (Munoz et al., 2005; Yan et al., 2014). However, such therapy is currently limited due to its limitations, such as ethical and moral concerns, the risk of immune rejection, the high risk of tumor formation, and technical difficulties (Song et al., 2024). Despite its limitations, stem cell therapy is still promising.

Neuron reprogramming involves the direct conversion of non-neuronal cells into neurons, providing a novel approach to neural repair. This therapy avoids ethical issues, immune rejection, and the risk of tumor formation associated with stem cell therapy. Astrocytes have attracted attention in neuronal reprogramming strategies. In addition to replenishing degenerating neurons, reprogramming astrocytes has the advantage of reducing glial scars (Liu et al., 2024c). The main mechanism of neuronal reprogramming is to reprogram target cells into pluripotent stem cells or neural progenitor cells by introducing multiple (POU class 5 homeobox 1, SRY-box transcription factor 2, v-myc avian myelocytomatosis viral oncogene homolog, kruppel-like factor 4) or single (distal-less homeobox 2 or SRY-box transcription factor 2) transcription factors, which in turn differentiate into various subtypes of neurons or glial cells (Wang et al., 2023c). Alternatively, genetic methods can be used to induce cells by overexpressing specific transcription factors (TFs) or microRNAs (Wan and Ding, 2023; Deng et al., 2025). Transcription factors and microRNAs induce reprogramming through similar yet different mechanisms. Transcription factors exert their effect by binding to target DNA, while microRNAs act after transcription but before translation. How to precisely regulate the production of the desired cells is the key to research in this type of therapy.

## Research Methods and Technological Advances

### Animal models

At present, animal models are mainly used to study NDDs, and different species or types of models have their own advantages and disadvantages. According to different pathological and pathophysiological factors, models can be divided into spontaneous, interventional, and transgenic models.

#### Spontaneous animal model of neurodegenerative diseases

Spontaneous animal models refer to disease models that occur naturally in experimental animals without any conscious artificial intervention. Spontaneous animal models are rare for various NDDs because these diseases rarely or never occur in many animals. Although these models have played a good role in simulating exogenous NDDs, a major limitation is that it takes a long time to wait for animals to develop spontaneous NDD-like changes. Spontaneous models of AD mainly include *Cercopithecidae* (e.g., *Macaca mulatta*) and *Canidae* (e.g., Beagle), which naturally exhibit age-related cognitive decline and some AD-like neuropathological features. Old monkeys show spontaneous cognitive impairment, which can be regarded as a spontaneous model of AD (Chen and Zhang, 2022). Only one case of spontaneous model animals of PD has been reported, and it is not widely used. For a long time, PD was previously considered a disease only happens in humans (Li et al., 2021b). Until 2021, a Chinese research team reported the first spontaneously occurring animal model of PD, which exhibited typical PD clinical symptoms, pharmacological responses, pathological features, and relevant genetic mutations (Li et al., 2021b). This finding suggests that monkeys have the potential to be an ideal model for PD. Some literature suggests that MS is unique to humans and that no other species’ spontaneous disease can better mimic human MS (Ransohoff, 2012). Some scholars have proposed using granulomatous meningoencephalomyelitis and Japanese macaque encephalomyelitis as spontaneous models of MS, but these models are less commonly used (Axthelm et al., 2011; Church et al., 2021). The spontaneous models of ALS include mouse and canine, which simulate the apparent spontaneity and gradual progression of MN loss observed in human ALS (Zhou et al., 2024b; **Additional Table 1**).

**Additional Table 1:** Common spontaneous models of neurodegenerative diseases

Disease	Animal	Model	Observed results/characteristics	Ethics approval number	Reference
Alzheimer's disease	Cercopithecidae	Macaca mulatta natural aging model	Shows cognitive decline and behavioral deficits around 20 years of age. Amyloid plaques mainly appear in the limbic cortex and prefrontal cortex. It can spontaneously present with neurofibrillary tau pathology, Aβ pathology, cholinergic dysfunction. The morphology and staging of plaques in its body are similar to those in humans. The amino acid sequence of Aβ in its body is the same as that of humans.	/	Braidy et al., 2015; Beckman and Morrison, 2021; McKean et al., 2021; Chen and Zhang, 2022
	Canidae	Canine cognitive dysfunction model	Exhibits symptoms such as disorientation, anxiety, and circadian rhythm changes with aging. Cortical atrophy and neuronal loss are observed in the cortex, hippocampus, and limbic system. The amino acid sequence of Aβ in its body is the same as that of humans. It can spontaneously present with Aβ pathology. No spontaneous neurofibrillary tau pathology. Lacks dense neuritic plaques and neurofibrillary tangles. The tau amino acid sequence is significantly different from that in humans.	/	Johnstone et al., 1991; Head, 2013; Braidy et al., 2015
Parkinson's disease	Cercopithecidae	Macaca fascicularis natural aging model	^1^Tremor, postural instability, and bradykinesia. ^1^Severe loss and morphological changes of dopaminergic neurons in the SN. Glial cell proliferation and activation. Presence of pathological α-syn, but no Lewy bodies.	^1^ Association for Assessment and Accreditation of Laboratory Animal Care accredited	Li et al., 2021b
Multiple sclerosis	Cercopithecidae	Japanese macaque encephalomyelitis	^1^Ataxia, paralysis, or significant visual impairment. ^2^Multifocal plaque-like demyelinating lesions observed. Lesions contain myelin oligodendrocyte glycoprotein, myelin basic protein and/or proteolipid protein-specific CD4^+^ T cells and CD8^+^ T cells. The central nervous system contains IL-17-positive active lesions.	^1^ Approved by the local Institutional Animal Care and Use Committee ^2^ Approved by the Oregon National Primate Research Center Institutional Animal Care and Use Committee	Blair et al., 2016; Govindan et al., 2021; Tagge et al., 2021
Amyotrophic lateral sclerosis	Muridae	The wobbler mouse	3-4 weeks after birth: weakness in the forelimbs, shaky gait, head tremor. The brain is rarely damaged. ^1^It shows motor neuron degeneration, glial proliferation, increased pro-inflammatory mediators and mitochondrial dysfunction. Death occurs within about 1 year.	^1^ NIH certificate # F16-00065 A5072-01	Meyer et al., 2023; Zhou et al., 2024b
		Progressive motor neuropathy mouse	Homozygotes develop hindlimb paralysis around 3 weeks after birth, forelimbs progressively affected. The skeletal muscle shows neurogenic atrophy, the distal motor axons are absent, and there is no demyelinating lesion. All homozygotes die at 6-7 weeks; heterozygotes are normal.	/	Schmalbruch et al., 1991
	Canine	Hereditary canine spinal muscular atrophy	Initial symptoms appear between 4 weeks and 4-6 months of age, weakness of proximal limb and trunk muscles.	/	Sumner, 2012; Zhou et al., 2024b
		Canine degenerative myelopathy canine	^1^Initial clinical symptoms are seen in dogs 8 years of age or older, with early symptoms of proprioceptive ataxia and spastic paralysis. Later, flaccid paralysis, extensive muscle atrophy, and respiratory dysfunction. Shows axonal degeneration, white matter demyelination, astrocyte proliferation, and SOD1 mutation. ^1^Shows elevated TDP-43 and SOD1 in the thoracic region of the spinal cord. ^1^Exosomal TDP-43, phosphorylated TDP-43, and SOD1 are elevated.	^1^ IACUC# 10077	Nardone et al., 2016; Pfeiffer et al., 2023; Espino and Miño, 2024

Aβ: Amyloid-beta; SODI: superoxide dismutase 1; TDP-43: TAR DNA-binding protein-43.

#### Interventional animal model of neurodegenerative diseases

The main intervention methods of the interventional model can be divided into chemical intervention, physical intervention, and biological intervention. The inducers of AD mainly include Aβ infusions, D-Galactose, Okadaic Acid, and Scopolamine. Each inducer can only induce some partial characteristic features of AD in experimental animals. β-Sitosterol-β-d-glucoside (BSSG) as a PD inducer has a progressive nature, allowing testing at a certain point in model development, and can reproduce disease progression without an initiating event (Van Kampen and Robertson, 2017; Monnot et al., 2025). The most widely used animal model for MS is experimental autoimmune encephalomyelitis (EAE), which is primarily induced in two ways: active immunization with myelin-derived proteins or peptides, and passive transfer of activated T cells (Dragic et al., 2020; Scherrer et al., 2025). EAE models can be induced in various animal species, including rats, mice, and non-human primates. The primary inducers for ALS models listed here are BSSG and L-β-methylamino-L-alanine (L-BMAA). The characteristics and comparisons of various animal models can be found in **Additional Table 2**.

**Additional Table 2:** Common intervention models of neurodegenerative diseases

Disease	Inducer	Intervention method	Common host animal	Observed results/ characteristics	Common intervention doses	Ethics approval number	Reference
Alzheimer's disease	Aβ infusions	Local injection (intracerebroventricular or hippocampus)	Rats, mice, and non-human primates	Induces Aβ aggregation, neuroinflammation, neuronal dysfunction, cognitive decline, and cholinergic impairment.	^1^Rats: Infuse Aβ_25-35_ into the hippocampus of each rat at a rate of 3.6 nmol/d for 14 days. ^1^Rats: Infuse Aβ_1-42_ into the hippocampus of each rat at a rate of 300 pmol/d for 14 days. ^1^Mice: Inject 1.2 μg of Aβ_1-42_ into the hippocampus of each mouse. ^1^Mice: Inject 3 nmol/3 μL of Aβ_25-35_ into the lateral ventricle of each mouse. ^1^Non-human primates (Maccaca fascicularis): They received intracerebroventricular injections of 10-100 μg of Aβ oligomers, administered once every 3 days for a period of up to 24 days.	^1^HSIACUC-20021 ^1^2015/229 ^1^KHMC-IACUC 15-031 ^1^02-2015 ^1^2011-039-OR	Forny-Germano et al., 2014; Elibol et al., 2020; Gutierrez et al., 2021; Kim and Cho, 2021; Hyun Yi et al., 2023; Ulaganathan and Pitchaimani, 2023; Yang et al., 2024
	D-Galactose	Intraperitoneal injection	Rats, mice	Anxiety, depression, cognitive impairment, motor deficits, Aβ aggregation, neuroinflammation, oxidative stress	^1,2,3^ Rats, ^4,5^mice: Inject 100-200 mg/kg/d intraperitoneally for 6-8 weeks.	^1^Animal experiments carried out were under the guidance of Ethical Committee Acts of Hubei University of Chinese Medicine ^2^Approved by Animal Ethics Committee of China Pharmaceutical University (Nanjing, China) ^3^No. SCXK (Ning) 2020-0001 ^4^Approved by the Animal Use and Care Committee of Fudan University ^5^2023010	Gao et al., 2016; Zhong et al., 2020; He et al., 2021; Rapaka et al., 2022; Zhang et al., 2023e; Nie et al., 2024; Samad et al., 2024
	Okadaic acid	Local injection (intracerebroventricular or hippocampal)	Rats, mice	Neuroinflammation, memory impairment, tau hyperphosphorylation, induction of oxidative stress, glial activation, lack of amyloid plaques	^1^^,2^Rats: Inject 200 ng into the intracerebroventricular space of rats in a single administration. ^1^Mice: Inject 200 ng into the hippocampus of mice in a single administration.	^1^IAEC/PCH01/2021-2022 ^2^IAEC 285 ^1^Approved by the Institutional Ethics Committee of China Medical University (Shenyang, China)	Kaushal et al., 2019; Pan et al., 2021; Dhapola et al., 2023; Dubey et al., 2024; Wang et al., 2024d
	Scopolamine	Intraperitoneal injection	Rats, mice	Cognitive impairment, oxidative stress, cholinergic dysfunction, neuroinflammation, induced amyloid plaque deposition, tau hyperphosphorylation.	^1^Rats, ^2,3^mice: Inject 1-2 mg/kg intraperitoneally.	^1^IR.MUMS.MEDICAL. REC.1399.788 FUTA/ ETH/21/20 ^2^No. K2018071 ^3^KNUST 0013	Akhtar et al., 2022; Assaran et al., 2022; Amoah et al., 2023; Dhapola et al., 2023; Wang et al., 2023a; Ogunsuyi et al., 2024
Parkinson's disease	BSSG	Oral administration	Rats	Progressive loss of DA neurons, bradykinesia, defective motor coordination, olfactory dysfunction, presence of large amounts of a-syn aggregates, microglial proliferation, cognitive deficits, no loss of spinal MNs	^1^Rats: Feed rats with food containing 3 mg/day of BSSG, 5 days a week for 16 weeks.	^1^# N13574/17	Van Kampen and Robertson, 2017; Monnot et al., 2025
	6-Hydroxydopamine	Local injection (SNpc, medial forebrain bundle, striatum)	Rats	Acute and severe degeneration of the DA system, anxiety and depressive-like behavior, mitochondrial dysfunction, free radical formation and oxidative stress	^1^^,2^Rats: Inject 10 pg at each site.	^1^Approved by the Experimental Animal Ethics Committee of The Fourth Affiliated Hospital of Guangxi Medical University. ^2^IR.TBZMED.AEC.1401.053	Yu et al., 2023; Azizifar et al., 2024; Kumari and Luthra, 2024
	MPTP	Systemic administration	Mice, non-human primates	Rapid neuronal death, retrograde degeneration, mitochondrial dysfunction, α-syn aggregation, degeneration of DA neurons, decreased 5-hydroxytryptamine levels, and motor dysfunction.	^1^Mice: Inject MPTP intraperitoneally using one of the following protocols: (1) 14-20 mg/kg every 2 hours for a total of 4 injections; or (2) 30 mg/kg/d for 5 consecutive days. ^1^Non-human primates (Macaca): Administer 0.5-1.5 mg/kg intravenously at weekly or longer intervals until stable parkinsonism develops.	^1^Approved by the Animal Ethics Committee of Jiangnan University. ^1^Approval by the Institutional Animal Care and Use Committee	Jackson-Lewis and Przedborski, 2007; Revuelta et al., 2012; Cui et al., 2023; Lal et al., 2024
Multiple sclerosis	Myelin-associated antigens or peptides (myelin basic protein, proteolipid protein, and MOG)	Subcutaneous injection	Rats, mice, and non-human primates	Ascending paralysis, weight loss, encephalitogenic T-cell responses, perivascular and subpial inflammatory infiltration, demyelinating lesions, glial cell proliferation, and axonal degeneration.	^1^Rats: Induced by subcutaneous injection of 0.1 mL encephalitogenic emulsion, which consisted of equal volumes of 50% (weight/volume) rat spinal cord homogenate in saline and Complete Freund's Adjuvant (1 mg/mL Mycobacterium tuberculosis). ^2^Mice (C57/BL6): Induced by subcutaneous injection 100 μg of MOG35-55 peptide emulsified in an equal volume of Complete Freund's Adjuvant supplemented with 400 μg of Mycobacterium tuberculosis H37 RA. Additionally, 0.3 μg of pertussis toxin was administered intraperitoneally. ^3^Non-human primates (Callithrix jacchus): Induced by intradermal injection of 400 μL emulsion, which consisted of 100 μg recombinant human MOG dissolved in 200 μL phosphate-buffered saline and emulsified with an equal volume of Incomplete Freund's Adjuvant, and immunizations were repeated approximately every 28 days until clinical symptoms were diagnosed.	^1^323-07-00622/2017-05 ^2^04/2019R ^3^DEC#674	Haanstra et al., 2013; Dragic et al., 2020; Scherrer et al., 2025
	Theiler's murine encephalomyelitis virus	Direct intracerebral infection	Mice	Transient limb flaccid paralysis, chronic inflammation, demyelination, neuronal apoptosis, activation of CD4^+^ and CD8^+^ T cells. Inflammatory demyelinating disease can be induced only in mice.	^1^Mice: Infected intracerebrally with 2 × 10^5^ plaque forming units of the DA strain of Theiler's murine encephalomyelitis virus.	^1^Approved by Institutional Animal Care and Use Committee of Kindai University Faculty of Medicine and Louisiana State University Health- Shreveport.	Procaccini et al., 2015; Lassmann and Bradi, 2017; Sato et al., 2022; Dedoni et al., 2023
	Encephalitogenic T cells	Intravenous or intraperitoneal injection	Mice	Neuroinflammation, axonal damage, infiltration of immune cells, but the passive transfer of CD4^+^ T-cells has no widespread focal primary demyelination	^1^Mice (C57Bl/6J): The lymph node cells from mice induced by MOG35-55 were expanded *in vitro*, and the activated T cells were intravenously transferred into syngeneic mice.	^1^Approved by the Washington University Animal Care Committee	Fritz and Zhao, 2001; Lees et al., 2008; Lassmann and Bradl, 2017
Amyotrophic lateral sclerosis	BSSG	Oral administration or local administration (in SN)	Rats, mice	Cognitive deficits, progressive motor dysfunction, increased astrocyte and microglial proliferation, oxidative stress, mitochondrial dysfunction	^1^Rats: Administer food containing 3 mg/day of BSSG, 5 days per week for 16 weeks. Mice: Fed with 0.5 g/d of cycad for up to 30 days.	^1^Approved by the University of Prince Edward Island Animal Care Committee /	’ Morrice et al., 2018; Bigelow et al., 2022; Lamichhane et al., 2025
	L-β-methylamino-L- alanine (L-BMAA)	Intraperitoneal injection or oral administration	Mice	TDP-43 accumulation in spinal MNs, glial activation and proliferation, degeneration and loss of MNs, motor coordination defects, cognitive decline.	^1,2^Mice (C57BL/6): (1) inject 30 mg/kg/day intraperitoneally for 30 consecutive days; or (2) incorporate 200-300 mg/kg/d into the food.	^'^Approved by the Animal Care Committee of "Federico II" University of Naples, Italy and Ministry of Health, Italy. ^2^Approved by the UC Irvine and Duke University Institutional Animal Care and Use Committees	Anzilotti et al., 2023; Arnold et al., 2023

Aβ: Amyloid-beta; BSSG: β-sitosterol-β-d-glucoside; DA: dopaminergic; MN: motor neuron; MOG: myelin oligodendrocyte glycoprotein; MPTP: 1-methyl-4-phenyl-1,2,3,6- tetrahydropyrldlne.

#### Transgenic animal models of neurodegenerative diseases

Transgenic models are a more commonly used animal model in research. There are many transgenic models for each disease, These models can selectively target one or multiple genes by knock-in, over-expression, or knock-out strategies. Since these models are induced by genetic manipulation, they are unlikely to represent true models of sporadic NDDs. There are many mouse transgenic models, including Tg2576 mouse (Klonarakis et al., 2022; Dhapola et al., 2023), M146L mouse (Duff et al., 1996; Barrow et al., 2000; Klonarakis et al., 2022; Collins and Greenfield, 2024), and L444P mouse (Dovonou et al., 2023; Mahoney-Crane et al., 2023; La Vitola et al., 2024). Additionally, multiple transgenic mice can be obtained through crossbreeding, such as the Tg2576×ApoE mouse model, which is obtained by crossbreeding Tg2576 mice with APOE knockout mice. Since many single transgenic animal models cannot meet the needs of multiple pathologies at the same time, multi-transgenic technology is therefore very important in the model construction process. Some single transgenic animal models are listed in **Additional Table 3**.

**Additional Table 3:** Common transgenic animal model of neurodegenerative diseases

Disease	Gene	Common animal model	Observed results/characteristics	Ethics approval number	Reference
Alzheimer's disease	*APP*	Tg2576 mouse	Tg2576 mice develop normally but exhibit age-related cognitive impairment and dendritic spine loss. Has elevated Aβ levels and amyloid plaque deposition, neuroinflammation, neurodegeneration, gliosis, and astrocytosis. Slow Aβ deposition.	/	Klonarakis et al., 2022; Dhapola et al., 2023
	*PSEN1*	M146L mouse	No pathological abnormalities within 2 years. Increased Aβ_42_ levels without plaque deposition. Calcium homeostasis disruption, and electrophysiological dysfunction.	/	Duff et al., 1996; Barrow et al., 2000; Klonarakis et al., 2022; Collins and Greenfield, 2024
	*MAPT*	P301L mouse	^1^Mild motor impairment, neuronal loss, tau hyperphosphorylation with conformational changes and aggregation. Calcifications and microstructural abnormalities. neurofibrillary tangles present. 1No axonal swelling. No brain atrophy. ^1^Average lifespan less than 12 months.	^1^ZH044/19	Terwel et al., 2005; Kindler et al., 2022; Wang et al., 2024d
Parkinson's disease	*LRRK2*	G2019S mouse	Behavioral abnormalities, impaired DA transmission, mitochondrial dysfunction, autophagy impairment, synaptic alterations, and tau hyperphosphorylation.	/	Dovonou et al., 2023; He et al., 2024
	*PINK1*	KO mouse	^1^Movement dysfunction, olfactory dysfunction, dysphagia, age- dependent deficits in DA levels. ^1^Defective dendritic spine maturation, reduced axonal synaptic vesicles, mitochondrial dysfunction.	^1^Approved by the Ethics Review Committee for Animal Experimentation of Central South University	Gao et al., 2022; He et al., 2024
	*DJ-1*	KO mouse	^1^Age-dependent movement deficits, abnormal DA metabolism, impaired dopaminergic function in the SN striatum. ^1^No significant loss of DA neurons and no significant neuropathology at 6 or 11 months of age.	^1^Approved by the Institutional Animal Care and Usage Committee of The University of Chicago	Chen et al., 2005; Dovonou et al., 2023
	*GBA1*	L444P mouse	^1^Homozygous mice show a-syn aggregation, astrogliosis, and a-syn accumulation in the striatum. ^1^Abnormal lysosomal storage in macrophage and formation of Gaucher cells. ^2^Heterozygous mice show mitochondrial dysfunction, increased ROS production. ^2,3^ An increase in a-syn pathology and spreading caused by a-syn injection. increased motor activity.	^1^Approved by the University of Massachusetts Medical School Animal Care and Use Committee ^2^Approved by the University of Alabama at Birmingham's Institutional Animal Care and Use Committee ^3^81-02.04.20 20.A169 81-02.04.2020. A277	Ginns et al., 2014; Dovonou et al., 2023; Mahoney-Crane et al., 2023; La Vitola et al., 2024
Multiple sclerosis	*IGH HLA*	IgH MOG mouse DR3 mouse	High frequency of anti-MOG antibody-secreting B cells in the peripheral immune repertoire. When immunized with MOG protein, T cells proliferate significantly and develop inflammatory lesions and demyelination in the CNS with mild to moderate clinical EAE.	/ /	Dedoni et al., 2023; Pressley et al., 2024 Pressley et al., 2024
	*TCR*	2D2 mouse	^1^Spontaneously develops EAE with slow progression with increased B cells in the blood, bone marrow, thymus, spleen, and lymph nodes. ^2^Alerted circadian rhythms. Spontaneous optic neuritis.	^1^Number Protocol 21-4 ^2^Approved by the Shanxi Committee on Ethics of Animal Research	Xue et al., 2018; Aulova et al., 2024
Amyotrophic lateral sclerosis	*SOD1*	G93A mouse	^1,2^Decreased motor function. ^1^Decline in maximal muscle strength of hindlimbs and combined hindlimbs and forelimbs and finally paralysis. ^1^Mitochondrial vacuolization. ^1,2^Defective oxidative phosphorylation. Neurodegeneration of spinal MNs and progressive motor deficits, cytoplasmic SOD1 aggregates, Golgi fragmentation, Lewy body-like inclusions, neurofilament-positive inclusions, gliosis, impaired calcium homeostasis, and MN degeneration.	^1^02/2023 Approved by the Animal Research Committee from the University of Seville (Spain) ^2^#17-061	López-Blanch et al., 2024; Belosludtseva et al., 2025; Gómez-Gálvez et al., 2025; Yang and Lee, 2025
	*TARDBP*	A315T mouse	^1^Gait abnormalities, rapid-onset early symptoms, shorter lifespan, different timelines of disease progression between males and females, progressive respiratory insufficiency, neuroinflammation in hypoglossal and phrenic motor nuclei, glial activation. ^2^Motor dysfunction, widespread neurodegeneration, muscle weakness, and atrophy. Recognition and memory impairments.	^1^Approved by Duke University Institutional Animal Care and Use Committee ^2^Approved by the Macquarie University animal care and ethics committee	Chan et al., 2020; Biswas et al., 2024; Lescouzères and Patten, 2024
	*FUS*	R521C mouse	Glial proliferation, neuromuscular junction defects, progressive neurodegeneration, and FUS accumulation. Develops significant neuronal loss in the spinal cord and muscle atrophy	/	Zhu et al., 2023a; López- Blanch et al., 2024)
	*C9ORF72*	KO mouse	^1^Mild or no motor deficits, lymphadenopathy and splenomegaly, T cell activation, immune system dysfunction, high titers of autoantibodies, increased expression of inflammatory cytokines. Macrophage immune dysregulation, lysosomal function defects.	^1^Approved by the Regeneron Pharmaceuticals Institutional Animal Care and Use Committee	Atanasio et al., 2016; Boivin et al., 2020; Jiang et al., 2022; Zhu et al., 2023a

APP: Amyloid beta precursor protein; Aβ: amyloid-beta; C9ORF72: chromosome 9 open reading frame 72; DA: dopaminergic; DJ-1: parkinsonism associated deglycase; FUS: fused in sarcoma; GBA: glucocerebrosidase; HLA: human leukocyte antigen; IGH: immunoglobulin heavy chain; KO: knockout; LRRK2: leucine rich repeat kinase 2; MAPT: microtubule- associated protein tau; PINK1: PTEN-induced kinase 1; PSEN1: presenilin 1; ROS: reactive oxygen species; SOD1: superoxide dismutase 1; TARDBP: TAR DNA-binding protein; TCR: T cell receptor; α-syn: α-synuclein.

### Application of multi-omics methods in neurodegenerative diseases

NDDs are usually complex diseases caused by multiple factors, involving interactions between genetics, environment, and other aspects. With the development of multi-omics technology, comprehensive analysis of data from genomics, transcriptomics, proteomics, and metabolomics provides new insights into understanding the mechanisms of these diseases, as well as early diagnosis and treatment.

Due to the rapid advances in GWAS and large-scale parallel sequencing technologies, numerous discoveries have been made in the genomics of NDDs (Sollis et al., 2023; Wu et al., 2024). GWAS focus on a large number of single nucleotide polymorphisms throughout the genome and analyze genetic variations associated with diseases (Aerqin et al., 2022). GWAS can also be used to analyze shared genetic risk factors across multiple diseases (Gibson et al., 2017). GWAS is crucial for understanding the fundamental causes of disease. For example, newly identified susceptibility loci for AD include *KIAA2013*, *SLC52A3*, and *TCN2* (Ge et al., 2024b). There are ethnic differences in the risk genes for AD, and the risk varies between ethnic groups (Miyashita et al., 2023). Genetic risk studies of PD have revealed that mutations in the PRKN cause autosomal recessive juvenile PD, while variants in *LRRK2* lead to autosomal dominant familial PD (Arya et al., 2024).

However, GWAS is primarily a population-based analysis, and its reference significance for individuals is limited. Therefore, next-generation sequencing techniques, including whole-genome sequencing and whole-exome sequencing, have emerged. These next-generation sequencing techniques make it possible to comprehensively analyze an individual’s genome (including coding and non-coding regions) and protein-coding regions to identify rare variants and structural abnormalities. Rare variations have a higher propensity to raise the risk of specific NDDs than common variants (Aerqin et al., 2022). This method has been applied in the analysis of some familial NDDs (Luo et al., 2021; Soudyab et al., 2022). Based on an extensive whole-exome sequencing screening, 12 new genetic variants were identified, and it was found that most of the early-onset PD patients in this study had *PRKN* gene variants (Soudyab et al., 2022). Another study based on whole-genome sequencing found a new missense mutation in *ACAA1* that contributes to early-onset AD and promotes Aβ pathology and cognitive impairment (Luo et al., 2021).

Transcriptomics primarily investigates RNA expression profiles, analyzing the types, expression levels, structures, and functions of transcripts. This method, mainly includes RNA sequencing and single-cell RNA sequencing (Liu and Song, 2025), can deepen the understanding of dysregulated pathways in NDDs and is the basis and starting point for the study of gene function and structure (Rexach et al., 2024). For example, in AD, new mechanisms of AD related to brain cell communication have been discovered (Zhang et al., 2023a). Another study identified C-X3-C motif chemokine receptor 1 (*CX3CR1*) as a novel peripheral biomarker for tauopathies and found that the expression and tau-related pathological alterations affect the abundance of human peripheral immune cells (Sirkis et al., 2023). In the course of research on ALS, the researchers found RNA processing dysregulation in ALS cases with *C9ORF72* repeat expansion (e.g., *FUS*, ataxin-2 (Prudencio et al., 2015). However, transcriptomic studies are limited in their ability to capture the dynamic changes and complex heterogeneity of the CNS, particularly in determining how gene expression alterations affect functional interactions between MNs and non-neuronal cells, and its correlation with the pathology of NDDs (Morello et al., 2020).

Proteomics can identify a large number of proteins in biological samples and study pathways in patients that may be related to diseases (Aerqin et al., 2022). Due to the complexity of NDDs, a single biomarker may not be sufficient to distinguish patients from the normal population. The development of proteomics enables the analysis of multiple proteins to identify novel biomarkers. The main research content of this method includes protein expression, protein interactions, and modifications. For example, proteomics studies have found that ATP synthase F1 subunit delta and calmodulin are downregulated in sALS (Raghunathan et al., 2022). In another study, midbrain dopaminergic axon degeneration induced by WD repeat domain 45 deficiency was found to be associated with lysophosphatidylcholine acyltransferase 1 and related phospholipid metabolism (Wang et al., 2024c). In AD, alterations in CSF neuronal pentraxin 2 have been observed and are considered a potential candidate biomarker for disease prognosis (Aerqin et al., 2022).

The primary focus of metabolomics is the measurement of small molecules known as metabolites in biological samples, including blood. And it is a method of omics that can capture real-time biological processes. The most commonly used tools for metabolic analysis include nuclear magnetic resonance spectroscopy, gas chromatography-mass spectrometry, and liquid chromatography-tandem mass spectrometry (Kim et al., 2024b). Metabolomics can provide a comprehensive understanding of dysfunctional metabolic pathways associated with NDDs and identify new biomarkers. For example, a recent study based on metabolomics found that the metabolic dysfunction of PD patients is mainly related to neurotransmitter metabolism and caffeine metabolism. It also suggests the possibility of norepinephrine and dihydroxyphenylacetic acid as biomarkers of DA neuron loss (Luo et al., 2024).

However, since protein expression is affected by a variety of post-transcriptional regulatory mechanisms, there is not always a direct linear relationship between protein levels and the expression of their corresponding genes. Therefore, there are certain limitations to the connection between proteomics and genomics. Metabolites, as the end products of biological processes, can reflect the terminal changes in physiological status. Unfortunately, there is a lack of knowledge regarding particular cellular and molecular regulatory mechanisms, which restricts a thorough comprehension of the disease (Kim et al., 2024b). Therefore, the role of single omics research in revealing the mechanism of disease is limited. Comprehensive analysis combining multiple omics can help discover new biomarkers and therapeutic targets, providing stronger assistance for clinical research (Kim et al., 2024b).

## Advances in Clinical Research

### Advances in clinical research of Alzheimer’s disease

Traditional AD medications can be categorized into two main types: acetylcholinesterase inhibitors and N-methyl-D-aspartic acid receptor antagonists (Zhang et al., 2024). In recent years, more and more drugs have been developed that are not limited to this scope. Among these drugs, clinical trial drugs targeting the immune system and inflammatory pathways can be typically classified into active immunization, passive immunization, and immunomodulatory drugs.

Active immunotherapies work by stimulating the body to produce antibodies targeting tau through vaccination (e.g., ACI-24.060, AV-1959). Passive immunizations can target Aβ, tau, or other related proteins using mAbs (e.g., Aducanumab, Donanemab, Gantenerumab, and Lecanemab). Immunomodulatory drugs primarily exert their effects through regulating enzyme activity (e.g., tyrosine kinase and Janus kinase), antagonizing receptors or channel proteins (e.g., calcium-activated potassium channels), and neutralizing inflammatory cytokines (e.g., TNF-α; **Additional Table 4**).

**Additional Table 4:** Typical drugs related to immune and inflammatory pathways for Alzheimer's disease

Drug	Type/target	Clinical trial	Lead sponsor
Aducanumab	Anti-amyloid monoclonal antibody	NCT04241068	Biogen
		NCT05310071	
Donanemab	Anti-amyloid monoclonal antibody	NCT04437511	Eli Lilly and Company
		NCT05026866	
		NCT05508789	
		NCT05738486	
Gantenerumab	Anti-amyloid monoclonal antibody	NCT01760005	Washington University School of Medicine
Lecanemab	Anti-amyloid monoclonal antibody	NCT01760005	Washington University School of Medicine
		NCT03887455	Eisai Inc.
		NCT04468659	
		NCT05269394	Washington University School of Medicine
Remternetug	Anti-amyloid monoclonal antibody	NCT05463731	Eli Lilly and Company
Solanezumab	Anti-amyloid monoclonal antibody	NCT01760005	Washington University School of Medicine
AV-1959	Anti-amyloid monoclonal antibody	NCT05642429	Institute for Molecular Medicine
Remternetug	Anti-amyloid monoclonal antibody	NCT04451408	Eli Lilly and Company
SHR-1707	Anti-amyloid monoclonal antibody	NCT06114745	Atridia Pty Ltd.
ABBV-916	Anti-amyloid antibody	NCT05291234	AbbVie
ALZ-101	Anti-Aβ protein vaccine	NCT05328115	Alzinova AB
ACI-24.060	Anti-Aβ protein vaccine	NCT05462106	AC Immune SA
Lecanemab	Anti-amyloid monoclonal antibody	NCT01767311	Eisai Inc.
Trontinemab	Monoclonal antibody directed at plaques and oligomers	NCT04639050	Hoffmann-La Roche
E2814	Anti-tau monoclonal antibody	NCT01760005	Washington University School of Medicine
		NCT05269394	
Bepranemab	Anti-tau monoclonal antibody	NCT04867616	UCB Biopharma SRL
E2814	Anti-tau monoclonal antibody	NCT04971733	Eisai Inc.
JNJ-63733657	Anti-tau monoclonal antibody	NCT04619420	Janssen Research & Development, LLC
MK-2214	Anti-tau monoclonal antibody	NCT05466422	Merck Sharp & Dohme LLC
APNmAb005	Anti-tau antibody	NCT05344989	APRINOIA Therapeutics, LLC
Masitinib	Inflammation	NCT05564169	AB Science
Semaglutide	Inflammation	NCT04777396	Novo Nordisk A/S
		NCT04777409	
		NCT05891496	
AL002	Inflammation	NCT04592874	Alector Inc.
		NCT05744401	
Astragalus	Inflammation	NCT05647473	Fujian Medical University Union Hospital
Bacillus Calmette-Guerin	Inflammation. Vaccine	NCT05004688	Steven E Arnold, MD
Baricitinib	Inflammation	NCT05189106	Massachusetts General Hospital
Dasatinib + Quercetin	Inflammation	NCT04685590	Wake Forest University Health Sciences
		NCT04785300	James L. Kirkland, MD, PhD
		NCT05422885	Lewis Lipsitz
Canakinumab	Inflammation	NCT04795466	Novartis Pharmaceuticals
Lenalidomide	Inflammation	NCT04032626	St. Joseph's Hospital and Medical Center, Phoenix
		NCT06177028	
L-Serine	Inflammation	NCT03062449	Aleksandra Stark
Montelukast buccal film	Inflammation	NCT03402503	IntelGenx Corp.
Pegipanermin	Inflammation	NCT05318976	Inmune Bio, Inc.
		NCT05522387	
Pepinemab	Inflammation	NCT04381468	Vaccinex Inc.
PrimeC	Inflammation	NCT06185543	NeuroSense Therapeutics Ltd.
Proleukin	Inflammation	NCT05468073	Center Hospitalier St Anne
Sargramostim	Inflammation	NCT04902703	University of Colorado, Denver
Senicapoc	Inflammation	NCT04804241	University of California, Davis
TB006	Inflammation	NCT05476783	TrueBinding, Inc.
Tdap	Inflammation	NCT05183516	Mindful Diagnostics and Therapeutics, LLC
Flos gossypii flavonoids	Inflammation	NCT05269173	Capital Medical University
Interleukin-2	Inflammation	NCT06096090	The Methodist Hospital Research Institute
Bacillus Calmette-Guerin	Inflammation	NCT06078891	Tamir Ben-Hur
CpG1018	Inflammation	NCT05606341	NYU Langone Health
Emtricitabine	Inflammation	NCT04500847	Butler Hospital
IBC-Ab002	Inflammation	NCT05551741	Immunobrain Checkpoint
Probiotic Blend Capsule	Inflammation and gut-brain axis	NCT06181513	University of Nicosia
GSK4527226	Anti-sortilin monoclonal antibody	NCT06079190	GlaxoSmithKline

Retrieved from ClinicalTrials.gov (accessed on April 11, 2025) and reference (Cummings et al., 2024).

Currently, many drugs have also been approved by the U.S. Food and Drug Administration (FDA) for marketing, including traditional treatments such as Donepezil, Galantamine, Rivastigmine, Memantine, and Namzaric, as well as new immune-related drugs such as Lecanemab, Donanemab, and Aducanumab. The FDA approved Lecanemab in 2023, and it is thought to be the best second-generation monoclonal antibody for Aβ protofibril immunodepletion (Vitek et al., 2023; Cheng et al., 2024). Donanemab exhibits selective binding to Aβ epitopes present in insoluble plaques, without interacting with soluble Aβ species (Vitek et al., 2023). In addition, Donanemab significantly slowed clinical progression in populations with tau pathology in other clinical trials (Sims et al., 2023). Unfortunately, current medications cannot address the need for cognitive impairments (Zhang et al., 2024), with the attrition rate for AD clinical trials remaining 98% (Cheng et al., 2024). Some drugs have been controversial since they were approved (Alexander et al., 2021; Huang et al., 2025a).

### Advances in clinical research of Parkinson’s disease

As recorded by ClinicalTrials.gov, there were 136 registered ongoing phase 1–3 trials evaluating pharmacological treatments for PD (as of January 31, 2024) (McFarthing et al., 2024). Among them, 76 trials (56%) are categorized as symptomatic treatment trials or disease-modifying therapy trials for PD (McFarthing et al., 2024). Among all immune- and inflammation-related clinical trial drugs targeting PD, they can also be categorized into active immunotherapy, passive immunotherapy, and anti-inflammatory therapies.

The active immunization vaccines mainly include PD01A, PD03A, and UB-312. PD01A and PD03A are two novel candidate vaccines containing short peptides as the antigenic component, designed to induce a sustained antibody response. The vast majority of adverse events are mild to moderate and are primarily related to injection site reactions, indicating good safety (Meissner et al., 2020). In clinical trials for PD, a significant increase in the antibody titer against α-syn was observed (Volc et al., 2020). UB‐312 is a synthetic α-syn peptide conjugated to a T helper peptide (Yu et al., 2022) capable of inducing a targeted B cell-mediated humoral response without T cell-mediated toxicity (Eijsvogel et al., 2024). Safety was found to be similar in all groups in clinical trials; most adverse events were mild and transient, and the treatment demonstrated significant efficacy against α-syn, supporting the further development of UB-312 (Volc et al., 2020; Eijsvogel et al., 2024).

Passive immunization drugs work by targeting α-syn, such as Prasinezumab, Cinpanemab, ABBV-0805, and PF-06456388. These drugs showed promising results in phase 1 clinical trials. However, it has recently been discovered that some mAbs that entered phase 2 clinical trials failed to demonstrate efficacy against α-syn (Pagano et al., 2022; Hutchison et al., 2024). Anti-inflammatory drugs targeting PD include Sargramostim, NE3107, Azathioprine, Montelukast, RO7486967, etc (Patel et al., 2024; **Additional Table 5**). The number of newly registered trials that target inflammation has significantly increased in recent years (McFarthing et al., 2024).

**Additional Table 5:** Typical drugs related to immune and inflammatory pathways for Parkinson's disease

Drug	Type	Clinical trial	Lead sponsor
PD01A	Active immunity vaccine	NCT06015841	AC Immune SA
PD03A	Active immunity vaccine	NCT02267434	Affiris AG
		NCT02758730	
		NCT02618941	
		NCT02216188	
		NCT01568099	
UB-312	Active immunity vaccine	NCT05634876	NYU Langone Health
		NCT04075318	United Neuroscience Ltd.
Prasinezumab	Anti-a-syn monoclonal antibody	NCT04777331	Hoffmann-La Roche
		NCT03100149	
BIIB054	Anti-a-syn monoclonal antibody	NCT03318523	Biogen
		NCT03716570	
		NCT02459886	
ABBV-0805	Anti-a-syn monoclonal antibody	NCT04127695	AbbVie
		NCT06671938	BioArctic AB
TAK-341	Anti-a-syn monoclonal antibody	NCT03272165	AstraZeneca
		NCT05526391	Takeda
		NCT04449484	
Lu AF82422	Anti-α-syn monoclonal antibody	NCT06258720	Lundbeck A/S
		NCT06706622	
		NCT05104476	
		NCT03611569	
VENT-02	Inflammation	NCT03659682	Ventus Therapeutics U.S., Inc.
Sulforaphane	Inflammation	NCT03958708	Central South University
Semaglutide	Inflammation. NLRP3 inhibitors	NCT06822517	Oslo University Hospital
Rifaximin	Inflammation	NCT05084365	Taipei Medical University Shuang Ho Hospital
		NCT06231563	University of California, San Francisco
Ketamine	Inflammation	NCT06816810	VA Office of Research and Development
Hydroxychloroquine	Inflammation	NCT03575195	Ottawa Hospital Research Institute
Cilostazol	Inflammation	NCT06612593	Ain Shams University
STEM-PD	Inflammation	NCT05635409	Region Skane

Retrieved from ClinicalTrials.gov (accessed on April 11, 2025).

As of April 11, 2025, all FDA-approved PD drugs include Cogentin, Eldepryl, Mirapex, Requip, Tasmar, Comtan, Stalevo, Symmetrel, Artane, Parcopa, Apokyn, Zelapar, Azilect, Neupro, Requip XL, Mirapex ER, Sinemet, Sinemet CR, Rytary, Duopa, Xadago, Gocovri, Inbrija, Osmolex, Nourianz, Ongentys, Kynmobi (Chopade et al., 2023), and Vyalev. Most of these drugs exert their therapeutic effects by modulating DA-related mechanisms (e.g., substitution and supplementation).

### Advances in clinical research of multiple sclerosis

In recent years, there have been three key therapeutic strategies for MS trials-the immune system (including active immunity, passive immunity, anti-inflammation), neuroprotection, and remyelination and repair (Chataway et al., 2024). Investigation of ClinicalTrials.gov found that there are relatively few active immunization trials in phase 1–3 clinical trials, mainly including Tovaxin Autologous T Cell Vaccine, Tolerogenic dendritic cells, NeuroVax, Bacille of Calmette-Guerin (**Additional Table 6**). All but one clinical trial has ended, and the results of some trials were not satisfactory (Fox et al., 2012). The clinical trial using Tolerogenic dendritic cells is expected to end on October 29, 2027.

**Additional Table 6:** Typical drugs related to immune and inflammatory pathways for multiple sclerosis

Drug	Type	Clinical trial	Lead sponsor
Tovaxin autologous T cell vaccine	Active immunity vaccine	NCT00587691	Opexa Therapeutics, Inc.
		NCT00245622	
Tolerogenic dendritic cells	Active immunity vaccine	NCT06715605	University Hospital, Antwerp
		NCT02618902	
NeuroVax	Active immunity vaccine	NCT02200718	Immune Response BioPharma, Inc.
		NCT02149706	
		NCT02057159	
Bacille of Calmette-Guerin	Active immunity vaccine	NCT00202410	S. Andrea Hospital
M-T412	Monoclonal antibody	NCT00004816	National Center for Research Resources
Daclizumab	Monoclonal antibody	NCT00001934	National Institute of Neurological Disorders and Stroke
	Monoclonal antibody	NCT00109161	PDL BioPharma, Inc.
		NCT00071838	National Institute of Neurological Disorders and Stroke
VX15/2503	Monoclonal antibody	NCT01764737	Vaccinex Inc.
VAY736	Monoclonal antibody	NCT02038049	Novartis Pharmaceuticals
Ublituximab	Monoclonal antibody	NCT02738775	TG Therapeutics, Inc.
		NCT04130997	
SAR441344 IV	Monoclonal antibody	NCT04879628	Sanofi
Rituximab	Monoclonal antibody	NCT01719159	Anders Svenningsson
		NCT04688788	Rigshospitalet, Denmark
rHIgM22	Monoclonal antibody	NCT01803867	Acorda Therapeutics
Ofatumumab	Monoclonal antibody	NCT01457924	GlaxoSmithKline
		NCT00640328	
		NCT03560739	Novartis Pharmaceuticals
		NCT04486716	
		NCT02792218	
		NCT03249714	
		NCT04510220	Brigham and Women's Hospital
		NCT06869785	
		NCT02792231	
Ocrelizumab	Monoclonal antibody	NCT02980042	University of Colorado, Denver
		NCT04544436	Hoffmann-La Roche
		NCT04548999	
Obexelimab	Monoclonal antibody	NCT06564311	Zenas BioPharma (USA), LLC
MOR103	Monoclonal antibody	NCT01517282	MorphoSys AG
LY2127399	Monoclonal antibody	NCT00882999	Eli Lilly and Company
GSK2618960	Monoclonal antibody	NCT01808482	GlaxoSmithKline
GNbAC1	Monoclonal antibody	NCT03239860	GeNeuro SA
		NCT03574428	GeNeuro Australia PTY Ltd
		NCT01639300	GeNeuro Innovation SAS
		NCT02782858	
Foralumab	Monoclonal antibody	NCT05029609	Tiziana Life Sciences Ltd.
		NCT06292923	
BIIB033	Monoclonal antibody	NCT01244139	Biogen
BCD-132	Monoclonal antibody	NCT03551275	Biocad
		NCT05385744	
AT-1501	Monoclonal antibody	NCT04322149	Anelixis Therapeutics, LLC
AIN457	Monoclonal antibody	NCT01433250	Novartis Pharmaceuticals
Active Comparator #1	Monoclonal antibody	NCT06894940	University of Pavia
ABT-874	Monoclonal antibody	NCT00086671	AbbVie (prior sponsor, Abbott)
Stem cell transplanataion	Inflammation	NCT00342134	National Institute of Neurological Disorders and Stroke
Simvastatin	Inflammation	NCT00429442	Anna Tsakiri
Methylprednisolone	Inflammation	NCT02784210	National Institute of Neurological Disorders and Stroke
		NCT01305837	Rigshospitalet, Denmark
KYV-101	Inflammation	NCT06451159	Bruce Cree
Epigallocatechin gallate	Inflammation	NCT00525668	Charite University, Berlin, Germany
BIIB091	Inflammation	NCT05798520	Biogen
ATA188	Inflammation	NCT03283826	Atara Biotherapeutics

Retrieved from ClinicalTrials.gov (accessed on April 11, 2025).

Passive immunity against MS mainly involves various mAbs, which target cell molecules (e.g., CD20 and CD100) and interleukins (e.g., IL-12 and IL-2). They exert their effect by regulating the function of immune cells and the level of inflammatory factors.

Drugs that target inflammation include Methylprednisolone, KYV-101, epigallocatechin-gallate, etc (**Additional Table 6**). KYV-101 is a type of CD19-targeted CAR-T cell therapy. It was found in its experiments that the production of intrathecal antibodies in the CSF after treatment with CAR-T cells was significantly reduced, showing good therapeutic potential (Fischbach et al., 2024).

A variety of drugs have been approved by the FDA to treat MS, including interferon-β, glatiramer acetate, Natalizumab, Fingolimod, Ozanimod, Teriflunomide, Dimethyl Fumarate, Ocrelizumab, Siponimod, Cladribine, Alemtuzumab, Ofatumumab. These drugs mainly reduce the attack of immune cells on the nervous system and inflammation by regulating the function of immune cells and acting on specific molecules.

### Advances in clinical research of amyotrophic lateral sclerosis

Investigation of ClinicalTrials.gov found that there have been no active immunotherapy (vaccines) trials for ALS in phases 1–3 clinical trials (accessed on April 11, 2025). However, in mouse experiments, tgG-DSE2lim and tgG-DSE5b have shown significant therapeutic effects (Zhao et al., 2019). Passive immunotherapy for ALS is also quite limited, including IC14, I10E, and AT-1501. IC14 is an anti-CD14 monoclonal antibody (Gelevski et al., 2023; **Table 6**). The adverse events that occurred during long-term treatment with IC14 were uncommon, mild, and self-limiting, demonstrating good safety. Moreover, a slower rate of Amyotrophic Lateral Sclerosis Functional Rating Scale-Revised decline was observed in this trial (Gelevski et al., 2023), and a reduction in neurofilament light chain levels was reported in another study (Henderson et al., 2021).

**Table 6:** Typical drugs related to immune and inflammatory pathways for amyotrophic lateral sclerosis

Drug	Type	Clinical trial	Lead sponsor
IC14	Anti-CD14 monoclonal antibody	NCT03474263	Implicit Bioscience
		NCT03487263	
		NCT03508453	
I10E	Human intravenous immunoglobulin	NCT01951924	Laboratoire français de Fractionnement et de Biotechnologies
AT-1501	Anti-CD40L monoclonal antibody	NCT04322149	Anelixis Therapeutics, LLC
NP001	Inflammation	NCT02794857	Neuraltus Pharmaceuticals, Inc.
Astaxanthin	Inflammation	NCT06429059	Duke University

Note: Retrieved from ClinicalTrials.gov (accessed on April 11, 2025).

ALS drugs that have been observed to have an effect on inflammation include NP001 and Astaxanthin (**Table 6**). NP001 is a small heterocyclic molecular regulator of inflammatory macrophages and monocytes. It slows the progression of Amyotrophic Lateral Sclerosis Functional Rating Scale-Revised and increases the overall survival of ALS patients by 4.8 months (Miller et al., 2022; Forrest et al., 2024). Astaxanthin is a fat-soluble carotenoid with anti-inflammatory, antioxidant, and anti-apoptotic effects. It has attracted attention for its protective effect against neurological diseases (Kandy et al., 2022; Si and Zhu, 2022). Phase 2 clinical trials are expected to be completed by May 1, 2025.

Therapeutic options for ALS remain relatively limited (Ashhurst et al., 2022). Drugs approved by the FDA for the treatment of ALS include Riluzole, Edaravone, Tofersen, and Relyvrio. The first ALS drugs approved by the FDA, Riluzole, inhibits glutamatergic neurotransmission in the CNS to produce neuroprotective effects (Fehlings et al., 2023). Among them, Relyvrio is a combination of Sodium Phenylbutyrate and Taurursodiol, which inhibits endoplasmic reticulum stress and reduces neuroinflammation through two mechanisms. Tofersen is the first treatment drug to be approved for a specific genetic mutation of ALS, which is of great significance.

## Limitations

Currently, many studies focus on the immune and inflammatory mechanisms of AD, PD, MS, and ALS. However, most of them are limited to a single disease. There are interconnections among different NDDs, and shared signaling pathways may mean that different diseases have the same or similar pathological basis. This may be of great significance for future treatments.

In addition, current treatment strategies seem to be limited to the use of biological or chemical methods (e.g., the use of antibodies, inhibitors). Perhaps physical therapy can be explored to avoid the immune risks and tumor risks associated with biological and chemical therapies. For example, some teams have used surgical methods to increase lymphatic drainage to improve NDDs (Li et al., 2024c).

## Summary and Outlook

Both genetic and cytological evidence suggest that the immune system (both peripheral and central) plays a crucial role in the genesis and development of NDDs. There is a complex network of interactions between them and between immune cells. The imbalance between immune cells will drive the development of NDDs. Microglia and astrocytes play a relatively important role in the pathogenesis and are a major focus of current research, particularly microglia-mediated NLRP3 inflammasome activation, T cell-mediated neuroinflammation, and multicellular interactions centered around glial cells. Different types of immune cells and their related signaling pathways, exhibit similar functions in various NDDs, but differ in pathological characteristics. In the pathophysiology of NDDs, the immune system has dual roles. In certain cases, pathological products such as Aβ and tau contribute to disease progression through a positive feedback mechanism. Currently, immunotherapy strategies have made some progress in clinical research. By targeting pathological proteins (e.g., Aβ, tau, and α-syn) and modulating immune-related targets (e.g., TREM2, NF-κB, and NLRP3 inflammasome), immunotherapy has opened new avenues for the treatment. However, despite the promising potential of immunotherapy demonstrated in laboratory studies and early clinical trials, its practical application still faces several challenges, including limited treatment options, side effects, unstable efficacy, and individual variability.

Future research should focus on several key areas to advance the application of immune and inflammatory therapies in NDDs. First, the interaction between immune cells and the nervous system needs to be explored in depth, especially the function of different immune cell types and their dynamic changes in exerting damage and protective effects. Research on the mechanisms of immune cells other than glial cells and T cells in NDDs should be increased to fill the gap in this field. In terms of treatment, personalized immunotherapy will become an important direction, especially given the high genetic polymorphism of the immune system. Under the premise of ensuring safety, candidate drugs with significant efficacy in preclinical studies can enter clinical trials. For example, TLR2 antagonists (e.g., CU-CPT22 (Li et al., 2024d)) can be used for clinical trials in early PD. However, given the particularity of the immune system, attention should be paid to immune risks and off-target effects when exploring treatment strategies. Although certain treatments (e.g., strategies targeting the gut microbiota (Hsu et al., 2023)) have efficacy in animal models, the intervention effects in clinical trials are still limited and have not yet met expectations. This might be related to the fact that most animal models are artificially induced, with relatively simple pathological mechanisms, while NDDs have complex etiologies and strong heterogeneity in humans, making it difficult for models to comprehensively simulate the characteristics of human diseases. In research, it is necessary to consider developing multi-factor induced composite models or developing humanized models (e.g., mouse models transplanted with human gut microbiota and using organoids (Gerasimova et al., 2025)) to enhance the simulation ability.

Although there have been gradual breakthroughs in drug research and development for NDDs, most drugs can only slow the progression of the disease. From preclinical research to clinical trials, the entire process faces challenges, including target selection, choice of animal models, target validation, biomarker identification, and adverse effect assessment (Xiao and Tan, 2025). More effective treatments can be sought by paying more attention to the above key processes. In addition, given the complexity of NDDs, a single therapeutic approach often fails to achieve satisfactory results. In individuals with PD, combining physical exercise with cognitive training may enhance overall cognitive performance, including memory, visuospatial abilities, and executive functions (Mantovani et al., 2024). When cognitive training is paired with transcranial direct current stimulation, it may further improve executive functioning and attention (Mantovani et al., 2024). Besides, some studies have found a link between whether or not the Tdap/Td vaccine is administered and the incidence of AD in epidemiological studies (Harris et al., 2023). These cases suggest that the combined use of drugs with different mechanisms of action, as well as combination therapies can be further explored. Besides, treatment approaches should not be limited to traditional strategies, and new therapeutic methods should be actively explored. Some physical therapies such as noninvasive brain stimulation, vagus nerve stimulation, exercise therapy, and the deep cervical lymphovenous anastomosis (Chen et al., 2025) reported in recent years are worthy of further investigation, but their efficacy and safety should be rigorously evaluated. Compared with before the operation, the MMSE score of patients after deep cervical lymphovenous anastomosis increased significantly, but long-term follow-up and large-scale clinical trials are still needed for verification (Chen et al., 2025). Besides, the abnormal accumulation of Aβ and tau in the brains of individuals with AD begins 10 to 20 years before the onset of dementia symptoms (Beason-Held et al., 2013). Non-motor symptoms may appear 10 to 20 years before the onset of motor symptoms in PD, and pathological α-syn aggregates can be observed in the gastrointestinal tract as early as 20 years before the diagnosis of PD (Hustad and Aasly, 2020). Approximately 51% of patients with radiologically isolated syndrome develop MS-like clinical symptoms within 10 years (Lebrun-Frénay et al., 2023). This suggests that researchers seek more effective and convenient early biomarkers and determine the optimal time point to start NDDs treatment. For research technology, researchers should take advantage of multi-omics technology and big data-based artificial intelligence. Artificial intelligence can play an important role in molecular design and synthesis, molecular selection, side effect and drug interaction prediction, etc. (Cheng et al., 2024).

## Additional files:

***Additional Table 1:*** *Common spontaneous models of neurodegenerative diseases.*

***Additional Table 2:*** *Common intervention models of neurodegenerative diseases.*

***Additional Table 3:*** *Common transgenic animal model of neurodegenerative diseases.*

***Additional Table 4:*** *Typical drugs related to immune and inflammatory pathways for Alzheimer’s disease.*

***Additional Table 5:*** *Typical drugs related to immune and inflammatory pathways for Parkinson’s disease.*

***Additional Table 6:*** *Typical drugs related to immune and inflammatory pathways for multiple sclerosis.*

## Data Availability

*All relevant data are within the paper and its Additional files*.
