# Corrigendum: Neurodegenerative diseases and immune system: From pathogenic mechanism to therapy

**DOI:** 10.4103/NRR.NRRONLINE-D-26-00258

**Published:** 2026-06-30

The affiliations for the first author, Yun Chen, and the corresponding author, Li Yu, of the article titled “Neurodegenerative diseases and immune system: From pathogenic mechanism to therapy,” published in issue 8, volume 21, 2026, in *Neural Regeneration Research*, were provided incorrectly. The corrected list of authors and their affiliations is as follows:

Yun Chen^1, #^, Ping Yin^2, #^, Qianqian Chen^2^, Yangyang Zhang^3^, Yangyi Tang^4^, Weifeng Jin^3, *^, Li Yu^1, 2, 5, *^

^1^The Fourth School of Clinical Medicine, Zhejiang Chinese Medical University, Hangzhou First People’s Hospital, Hangzhou, Zhejiang Province, China; ^2^School of Basic Medical Sciences, Zhejiang Chinese Medical University, Hangzhou, Zhejiang Province, China; ^3^School of Pharmaceutical Sciences, Zhejiang Chinese Medical University, Hangzhou, Zhejiang Province, China; ^4^The First School of Clinical Medicine, Zhejiang Chinese Medical University, Hangzhou, Zhejiang Province, China; ^5^Key Laboratory of Drug Safety Evaluation and Research of Zhejiang Province, Center of Safety Evaluation and Research, Hangzhou Medical College, Hangzhou, Zhejiang Province, China

The online version of the original article can be found under doi: 10.4103/NRR.NRR-D-25-00274.
